# Suppression of 6-Hydroxydopamine-Induced Oxidative Stress by Hyperoside Via Activation of Nrf2/HO-1 Signaling in Dopaminergic Neurons

**DOI:** 10.3390/ijms20235832

**Published:** 2019-11-20

**Authors:** Seung-Hwan Kwon, Seoung Rak Lee, Yong Joo Park, Moonjin Ra, Yongjun Lee, Changhyun Pang, Ki Hyun Kim

**Affiliations:** 1 Neuroregeneration and Stem Cell Programs, Institute for Cell Engineering, The Johns Hopkins University School of Medicine, Baltimore, MD 21205, USA.; skwon28@jhmi.edu; 2 Department of Neurology, The Johns Hopkins University School of Medicine, Baltimore, MD 21205, USA; 3 School of Pharmacy, Sungkyunkwan University, Suwon 16419, Korea; davidseoungrak@gmail.com (S.R.L.); pyj084@msn.com (Y.J.P.); 4 Hongcheon Institute of Medicinal Herb, 101 Yeonbongri, Hongcheon 25142, Korea; ramj90@himh.re.kr (M.R.); leerayongjun@gmail.com (Y.L.); 5 School of Chemical Engineering, Sungkyunkwan University, Suwon 16419, Korea; 6 SKKU Advanced Institute of Nanotechnology Sungkyunkwan University, Suwon 16419, Korea

**Keywords:** hyperoside, *Acer tegmentosum*, 6-hydroxydopamine, nuclear factor erythroid 2-related factor 2, heme oxygenase-1, Parkinson′s disease

## Abstract

In our ongoing research to discover natural products with neuroprotective effects, hyperoside (quercetin 3-*O*-galactoside) was isolated from *Acer tegmentosum*, which has been used in Korean traditional medicine to treat liver-related disorders. Here, we demonstrated that hyperoside protects cultured dopaminergic neurons from death via reactive oxygen species (ROS)-dependent mechanisms, although other relevant mechanisms of hyperoside activity remain largely uncharacterized. For the first time, we investigated the neuroprotective effects of hyperoside on 6-hydroxydopamine (6-OHDA)-induced neurotoxicity in neurons, and the possible underlying mechanisms. Hyperoside significantly ameliorated the loss of neuronal cell viability, lactate dehydrogenase release, excessive ROS accumulation and mitochondrial membrane potential dysfunction associated with 6-OHDA-induced neurotoxicity. Furthermore, hyperoside treatment activated the nuclear erythroid 2-related factor 2 (Nrf2), an upstream molecule of heme oxygenase-1 (HO-1). Hyperoside also induced the expression of HO-1, an antioxidant response gene. Remarkably, we found that the neuroprotective effects of hyperoside were weakened by an Nrf2 small interfering RNA, which blocked the ability of hyperoside to inhibit neuronal death, indicating the vital role of HO-1. Overall, we show that hyperoside, via the induction of Nrf2-dependent HO-1 activation, suppresses neuronal death caused by 6-OHDA-induced oxidative stress. Moreover, Nrf2-dependent HO-1 signaling activation represents a potential preventive and therapeutic target in Parkinson′s disease management.

## 1. Introduction

The neuropathology of Parkinson′s disease (PD) is characterized by the loss of dopaminergic neurons in the substantia nigra pars compacta (SNpc), as a result of many contributing factors, including oxidative stress-mediated, mitochondrial dysfunction, environmental toxins and inflammation, ultimately leading to the death of neuronal cells [1]. Accordingly, we selected 6-hydroxydopamine (6-OHDA)-induced neurotoxicity and oxidative stress to mimic the neuropathological symptoms of PD in an in vitro cellular model [2]. Oxidative stress is associated with PD dementia and dementia with Lewy bodies, and thus is considered to be responsible for significant central events in the process of PD pathogenesis [3].

Thus, pharmacological or nutritional interventions to reduce oxidative stress may potentially ameliorate oxidative stress-related neurodegenerative diseases, including Alzheimer′s disease (AD), amyotrophic lateral sclerosis (ALS) and PD [3,4,5]. Oxidative stress can be defined as an imbalance between cellular antioxidant defense systems and the endogenous or exogenous pro-oxidant burden [6]. Excessive production and the accumulation of reactive oxygen species (ROS) induced by 6-OHDA can lead to DNA cleavage, protein oxidation and lipid peroxidation, ultimately resulting in cellular dysfunction and apoptosis [7]. Controlling ROS production and scavenging may be therapeutically beneficial in the management of PD [8].

As part of our ongoing studies to explore structurally and pharmacologically-active, unique natural products from diverse, natural sources [9,10,11,12,13], we have recently focused on the identification of neuroprotective compounds [13]. Recently, daidzein-7-*α*-L-rhamnopyranoside, identified from the fungus-growing termite-associated *Streptomyces* sp. RB1, was found to prevent glutamate-induced hippocampal neuronal cell death via its blocking of intracellular ROS accumulation in HT-22 cells [13]. In the present study, we investigated *Acer tegmentosum* Maxim (Aceraceae), which has been used in Korean traditional medicine to treat liver-related disorders, including hepatitis, liver cancer and hepatic cirrhosis, as well as to detoxify the liver. [14]. Previous phytochemical investigations of *A. tegmentosum* have revealed that its extracts contain various pharmacological metabolites, including flavonoids, lignans, and phenolic compounds [15,16]. In a recent study, we successfully isolated and identified a new phenolic compound, isoamericanoic acid B, and nine phenolic compounds from the bark of *A. tegmentosum* [17]. Intriguingly, we observed that a coumarin glycoside, fraxin, isolated from *A. tegmentosum*, displayed potent hepatoprotective effects via the nuclear factor erythroid 2-related factor 2 (Nrf2)-mediated antioxidant enzyme system [18].

Nrf2 is a transcription factor that, when activated, translocates to the nucleus to activate homeostatic cell defenses in response to oxidative stress, initiates phase II enzyme expression, such as heme oxygenase-1 (HO-1) and NADPH quinone oxidoreductase 1 (NQO1), which are antioxidant response elements (ARE), and mitigates the pathogenesis of PD [19]. In PD, Nrf2 is localized in the nucleus; however, insufficient Nrf2 activation has been observed in the brains of PD patients, compared to healthy individuals [20]. In animal models, Nrf2-deficient mice are highly susceptible to neurotoxin-induced dopaminergic degeneration [21,22]. Among the various known cytoprotective enzymes, HO-1 has received considerable attention [23,24]. Indeed, several recent studies have demonstrated that an activation of the Nrf2-dependent HO-1 pathway ameliorates the neurotoxicity in oxidative stress-induced neuronal cells [25,26]. Moreover, induced phase II enzymes, such as HO-1, have the potential to improve cellular antioxidant capacity and protect against oxidative injury; thus, the induction of HO-1 is a common feature in several neurodegenerative diseases, such as AD and PD [27,28,29]. Consequently, the activation of Nrf-2-mediated HO-1 induction is considered a potential therapeutic target in the management of PD [30,31].

In line with this evidence, we investigated potential neuroprotective metabolites associated with Nrf2-mediated antioxidants, such as HO-1 from an extract of *A. tegmentosum.* Column chromatographic separation of the EtOAc-soluble fraction of the extract led to the isolation of 15 compounds, followed by high-performance liquid chromatography (HPLC) purification. In this study, cell-based protection screening was first performed to evaluate the neuroprotective effects of the isolated compounds **1**–**15** using human dopaminergic neuroblastoma SH-SY5Y cells. 

To elucidate the underlying neuroprotection mechanism, we studied the mechanism of action of the most active compound **5**, hyperoside (quercetin 3-*O*-galactoside), on 6-OHDA-induced oxidative stress in human SH-SY5Y dopaminergic neurons. We also investigated whether hyperoside activates Nrf2-dependent signaling, which induces HO-1 upregulation if it is downstream of the ARE-responsive proteins. Here, we describe, in detail, the neuroprotective effects of hyperoside via the activation of Nrf2/HO-1 signaling in human dopaminergic neuroblastoma SH-SY5Y cells.

## 2. Results

### 2.1. Isolation and Structural Identification of Compounds

**Figure 1:**
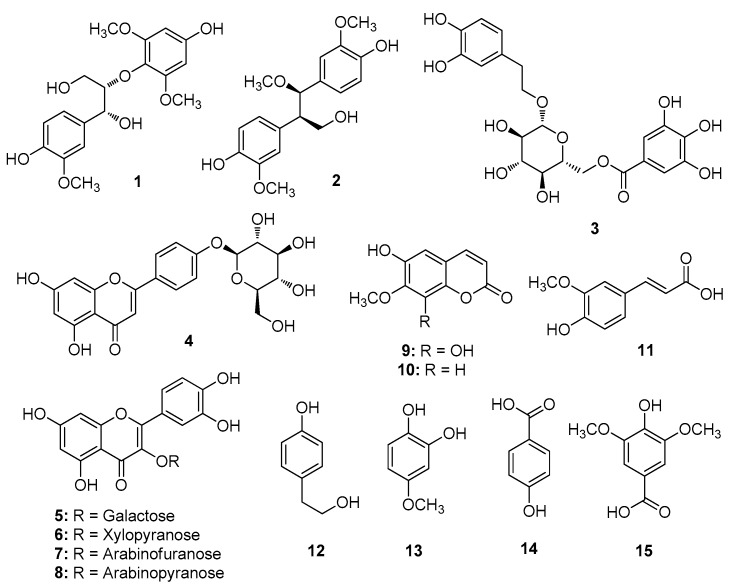
Chemical structures of compounds (**1**–**15**) isolated from *A. tegmentosum* bark.

Dried and pulverized *A. tegmentosum* bark was extracted with water at 90 °C and then filtered. The filtrate was concentrated under vacuum to obtain a crude aqueous extract and then solvent-partitioned with hexane, dichloromethane (CH_2_Cl_2_), ethyl acetate (EtOAc) and *n*-butanol (*n*-BuOH) to obtain four main fractions. Based on LC/MS analysis and preliminary proton nuclear magnetic resonance (^1^H NMR) spectroscopic analysis, the EtOAc-soluble fraction was selected to identify the bioactive constituents. Fractionation and purification following repeated column chromatography and semi-preparative HPLC of the EtOAc fraction resulted in the isolation of 15 phenolic compounds (**1**–**15**) (Figure 1).

The isolated compounds were identified as (7*S*,8*R*)-1′,4-dihydroxy-3,3′,5′-trimethoxy-7′,8′,9′-trinor-8,4′-oxyneoligna-7,9-diol (**1**) [32], 4,4′-((1*R*,2*R*)-3-hydroxy-1-methoxypropane-1,2-diyl)-bis-2-methoxyphenol (**2**) [33], 1-*O*-β-d-(6′′-*O*-galloyl)-glucopyranoside (**3**) [34], naringenin-4′-*O*-β-d-glucopyranoside (**4**) [35], hyperoside (**5**) [36], quercetin 3-β-d-xylopyranoside (**6**) [37], avicularin (**7**) [38], guaijaverin (**8**) [39], 6,8-dihydroxy-7-methoxy-2*H*-1-benzopyran-2-one (**9**) [40], 6-hydroxy-7-methoxy-2*H*-1-benzopyran-2-one (**10**) [41], (*E*)-ferulic acid (**11**) [42], 4-hydroxy-benzeneethanol (**12**) [43], 2-hydroxy-4-methoxyphenol (**13**) [44], 4-hydroxy-benzoic acid (**14**) [45] and syringic acid (**15**) [46] by comparing their NMR spectroscopic and physical data with those reported in the literature, and by measuring their specific rotations as well as via LC/MS analysis.

### 2.2. Neuroprotective Activity Screening of the Isolated Compounds Using a Cell Viability Assay

First, to identify the compounds with the highest neuroprotective effect, from a total of 15 compounds, on 6-OHDA-induced cell toxicity in SH-SY5Y cells, we examined cell viability using the MTT assay. Cells were pretreated with compounds (0, 0.25, 0.5, 1, 2.5, 5, or 10 μM) or NAC (as an antioxidant reference drug at 0.5, 1, 2.5, or 5 mM) for 4 h prior to exposure to 200 μM 6-OHDA for 24 h. Exposure to 200 μM 6-OHDA induced loss of cell viability (approximately 50%) after 24 h treatment (Figure S1). Among the compounds tested, nine compounds (**1**, **2**, **3**, **5**, **6**, **7**, **9**, **11** and **13**) significantly protected cells from the loss of viability induced by 6-OHDA. 

These compounds showed preventive effects in a relatively narrow effective concentration range against 6-OHDA-induced cell toxicity, i.e., at 2.5, 5 and 10 μM. Interestingly, compound **5** (hyperoside) showed the highest neuroprotective effect on 6-OHDA-induced cell toxicity at low concentrations, i.e., 0.5, 1 and 2 μM, compared to the other compounds. The following in vitro MTT assay showed that hyperoside has the most potently protective cells with IC_50_ of 0.86 μM among the isolated compounds **1**–**15**. Besides, the neuroprotective effect of hyperoside was stronger than that of the reference drug, NAC.

### 2.3. Hyperoside Inhibits 6-OHDA-Induced Cytotoxicity in SH-SY5Y Cells

**Figure 2:**
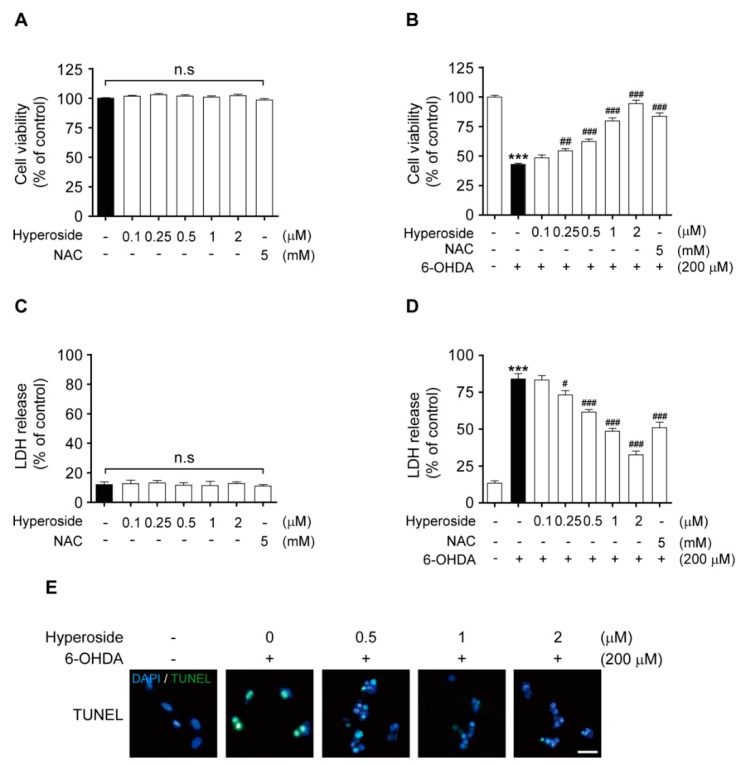
Effects of hyperoside on SH-SY5Y cells (**A** and **C**). Cells were pretreated with the indicated concentrations of hyperoside or *N*-acetylcysteine (NAC) for 24 h. Hyperoside protects against 6-hydroxydopamine (6-OHDA)-induced cell death (**B**) and lactate dehydrogenase (LDH) release (**D**) in SH-SY5Y cells. Cells were pretreated with the hyperoside (0.1–2 μM) or NAC (5 mM) for 4 h and then exposed to 200 μM 6-OHDA for 24 h. Error bars indicate the mean ± the standard error of the mean (SEM) (*n* = 6). Hyperoside prevents 6-OHDA-induced DNA fragmentation (**E**). DNA fragmentation was assayed using terminal deoxynucleotidyl transferase dUTP nick end labeling (TUNEL) staining. Cells were pretreated with the hyperoside (0.5–2 μM) for 4 h and then exposed to 200 μM 6-OHDA for 24 h. Images shown are representative of three experiments and visualized by fluorescence microscopy (20×). *** *p <* 0.001 vs. the control group. ^#^ *p* < 0.05, ^##^ *p* < 0.01 and ^###^ *p* < 0.001 vs. the 6-OHDA-treated group. n.s.: not significant. Scale bar: 200 μm.

To determine whether the neuroprotective effects of hyperoside (**5**) abrogate 6-OHDA-induced cell toxicity, we initially evaluated the cell viability and LDH release after pretreatment with the indicated concentrations of hyperoside (**5**) or NAC (0.1, 0.25, 0.5, 1 and 2 μM or 5 mM, respectively, for 4 h). Further, 200 μM 6-OHDA was used to induce cell injury in all subsequent experiments. Hyperoside and NAC did not induce cytotoxicity in SH-SY5Y cells at concentrations of 0.1, 0.25, 0.5, 1 and 2 μM or 5 mM, respectively (Figure 2A,C); thus, these concentrations were employed in subsequent experiments. Moreover, we considered it safe to use the respective concentrations of hyperoside for further studies. Next, the concentration-dependent neuroprotective effect of hyperoside (**5**) was determined using the MTT assay. Specifically, cells were pretreated with 0.1, 0.25, 0.5, 1 and 2 μM hyperoside or 5 mM NAC for 4 h prior to exposure to 200 μM 6-OHDA for 24 h. As shown in Figure 2B, pretreatment of cells with 0.25, 0.5, 1 and 2 μM hyperoside significantly inhibited the 6-OHDA-induced reduction in cell viability (*p* < 0.01 and *p* < 0.001, respectively). Furthermore, cells pretreated with 0.25, 0.5, 1 and 2 μM hyperoside showed significantly reduced 6-OHDA-induced LDH release, compared to control cells treated with 6-OHDA alone (Figure 2D, *p* < 0.05 and *p* < 0.001, respectively). To observe the neuroprotective effect of hyperoside on 6-OHDA-induced cell death and DNA fragmentation, we performed a TUNEL staining assay. In representative images, this TUNEL staining revealed significant DNA fragmentation after exposure to 6-OHDA, whereas pretreatment with hyperoside (**5**) significantly prevented DNA fragmentation (Figure 2E).

### 2.4. Hyperoside Prevents 6-OHDA-Induced Intracellular ROS Accumulation and Mitochondrial Membrane Potential Dysfunction in SH-SY5Y Cells

**Figure 3:**
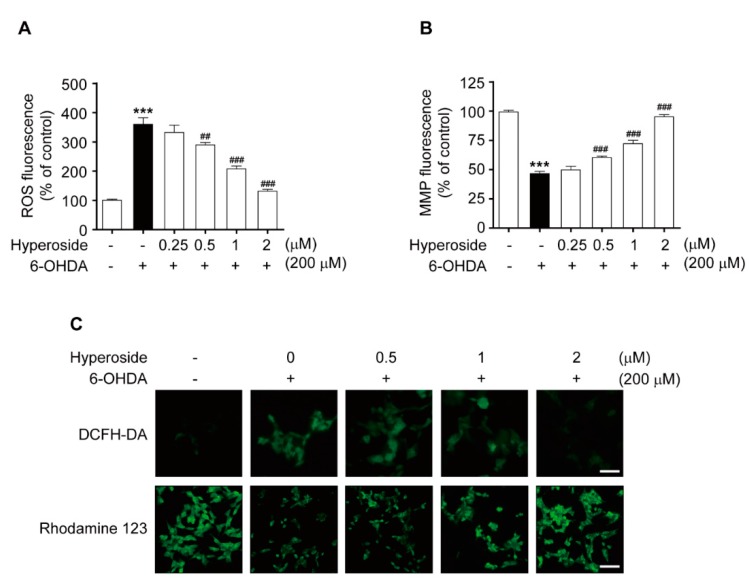
Hyperoside prevents 6-OHDA-induced intracellular Reactive Oxygen Species (ROS) accumulation (**A**,**C**, upper) and mitochondrial membrane potential (MMP) dysfunction (**B**,**C**, bottom) in SH-SY5Y cells. Representative images were observed under fluorescence microscopy (20×). Cells were pretreated with the hyperoside (0.25–2 μM) for 4 h and then exposed to 200 μM 6-OHDA for 1 or 24 h. Error bars indicate the mean ± SEM (*n* = 6). Images shown are representative of three experiments and visualized by fluorescence microscopy (40×). *** *p <* 0.001 vs. control group. ^##^ *p* < 0.01 and ^###^ *p* < 0.001 vs. the 6-OHDA-treated group. Scale bar: 200 μm.

Next, we investigated intracellular ROS accumulation and mitochondrial membrane potential (MMP) dysfunction, which are well-known initiators of the oxidative stress that induces cell injury. Treatment with 200 μM 6-OHDA significantly increased the intracellular ROS, compared to the control (Figure 3A, *p* < 0.001 and Figure 3C, upper); however, the 6-OHDA-mediated increase in intracellular ROS was significantly prevented by pretreatment with hyperoside at 0.5, 1 and 2 μM (*p* < 0.01 and *p* < 0.001, respectively). Conversely, treatment with 200 μM 6-OHDA significantly decreased this MMP, compared to the control (Figure 3B, *p* < 0.001 and Figure 3C, bottom); however, the 6-OHDA-mediated decrease in intracellular MMP was significantly prevented by pretreatment with hyperoside (**5**) at 0.5, 1, and 2 μM (*p* < 0.001, respectively).

### 2.5. Hyperoside-Mediated Activation of Nrf2 Occurred in a Time- and Concentration-Dependent Manner in SH-SY5Y Cells

**Figure 4:**
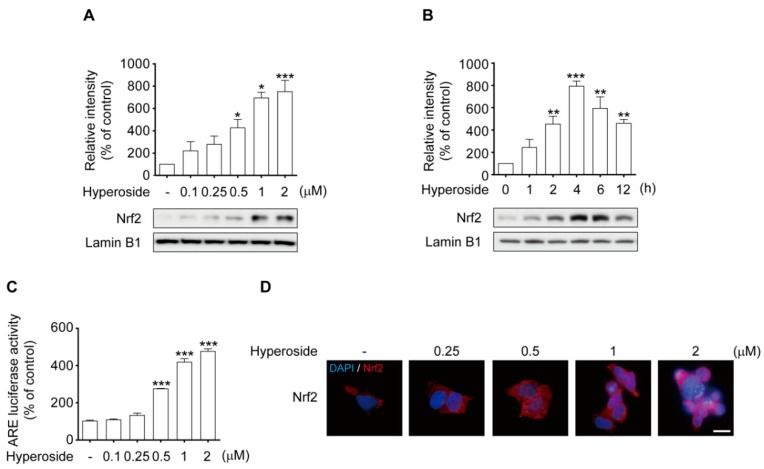
Hyperoside activates nuclear translocation of Nrf2 in SH-SY5Y cells. Hyperoside-induced Nrf2 activation is of a concentration- (**A**) and time-dependent (**B**) manner in SH-SY5Y cells. Cells were treated with hyperoside at the indicated times (0–12 h) and concentrations (0.1–2 μM), after which nuclear fractions were prepared to analyze the distribution of Nrf2. Levels of Nrf2 and laminin B1 were evaluated by Western blot analysis. Cells were transiently transfected with an ARE reporter plasmid construct and then treated with the indicated concentrations of hyperoside for 4 h. Equal amounts of cell extracts were assayed for dual-luciferase activity (**C**). Nrf2 was detected using an anti-Nrf2 antibody and a Texas red^®^-conjugated secondary antibody. Nuclei were stained with Hoechst 33258. Images shown are representative of three experiments and were captured using a fluorescence microscope (**D**, 100× magnification). Error bars indicate the mean ± SEM (*n = 3*). * *p* < 0.05, ** *p* < 0.01, and *** *p* < 0.001 vs. control group. Scale bar: 50 μm.

To examine whether hyperoside (**5**) induces the HO-1 transcriptional signaling pathway, which is directly linked to Nrf2-dependent activation, we used both western blot analysis and immunostaining. Treatment with hyperoside induced significant nuclear translocation of Nrf2 in both a concentration-dependent (Figure 4A, *p* < 0.05 and *p* < 0.001) and time-dependent (Figure 4B, *p* < 0.01 and *p* < 0.001) manner. Similarly, after treatment with 2 μM hyperoside, the nuclear protein levels of Nrf2 were significantly increased for 1 h, peaked at 4 h, and then decreased at 6 h. Based on these results, treatment with 2 μM hyperoside for 4 h was used to induce nuclear translocation of Nrf2 in subsequent experiments. In an attempt to determine whether induction of HO-1 by hyperoside (**5**) was indeed responsible via the activation of the ARE-binding ability of Nrf2, the cells were transfected with luciferase reporters under the control of the ARE promoter. As shown in Figure 4C, the transcriptional activity of ARE was significantly increased by treatment with hyperoside in a concentration-dependent manner, compared to the control (*p* < 0.001, respectively). Representative images reveal the nuclear inclusion of Nrf2 in cells treated with hyperoside (Figure 4D). As expected, pretreatment with hyperoside (**5**) activated the nuclear translocation of Nrf2 in the cells.

### 2.6. Hyperoside Induced the Expression of HO-1 in a Time and Concentration-Dependent Manner in SH-SY5Y Cells

**Figure 5:**
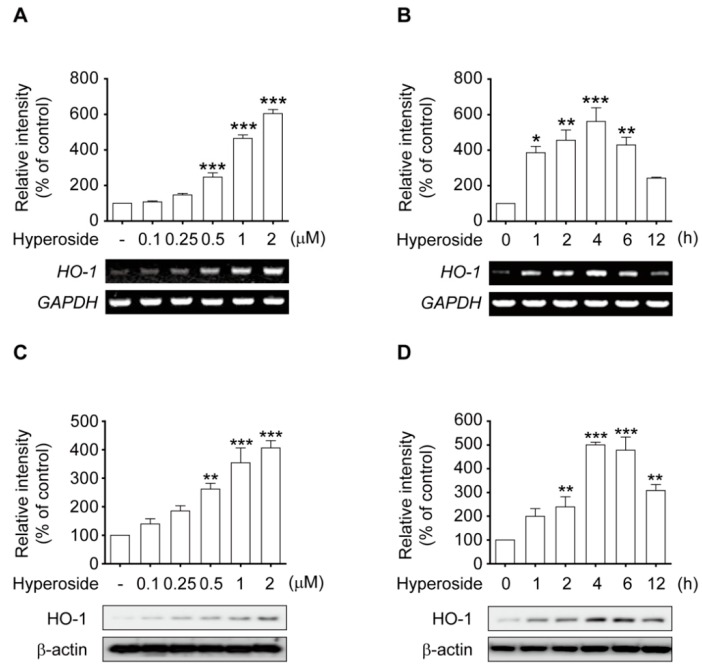
Hyperoside activates Nrf2-mediated HO-1 transcriptional induction in SH-SY5Y cells. Treatment with hyperoside induced the expression of HO-1 in a concentration-dependent manner (**A**,**C**). Cells were treated with hyperoside (0.1–2 μM) for 4 h or 6 h, and total mRNA and protein expression was analyzed by RT-PCR and Western blot analysis, respectively. Hyperoside induced the expression of HO-1 in a time-dependent manner (**B**,**D**). Cells were treated with 2 μM hyperoside for 0, 1, 2, 4, 6 and 12 h, and the total RNA and protein expression was analyzed by RT-PCR and Western blot analysis. Expression levels of HO-1, β-actin and glyceraldehyde 3-phosphate dehydrogenase (GAPDH) were evaluated by Western blotting and RT-PCR analysis. Error bars indicate the mean ± SEM (*n = 3*). * *p* < 0.05, ** *p* < 0.01, and *** *p* < 0.001 vs. the control group.

To determine whether the observed neuroprotective effects of hyperoside lead to the activation of the Nrf2-ARE pathway in SH-SY5Y cells, we evaluated both the protein and mRNA levels of HO-1, a downstream target of Nrf2, by Western blotting and RT-PCR analysis. Treatment with hyperoside (**5**) elicited a concentration-dependent (Figure 5A,C) and time-dependent (Figure 5B,D) increase in the induction of HO-1 protein and mRNA. Thus, cells were treated with 2 μM hyperoside for 4 or 6 h to induce HO-1 protein and mRNA expression in the subsequent experiments.

### 2.7. Nrf2 Gene Knockdown Eliminated the Neuroprotective Effects of Hyperoside on Nrf2-Mediated HO-1 Transcriptional Induction

**Figure 6:**
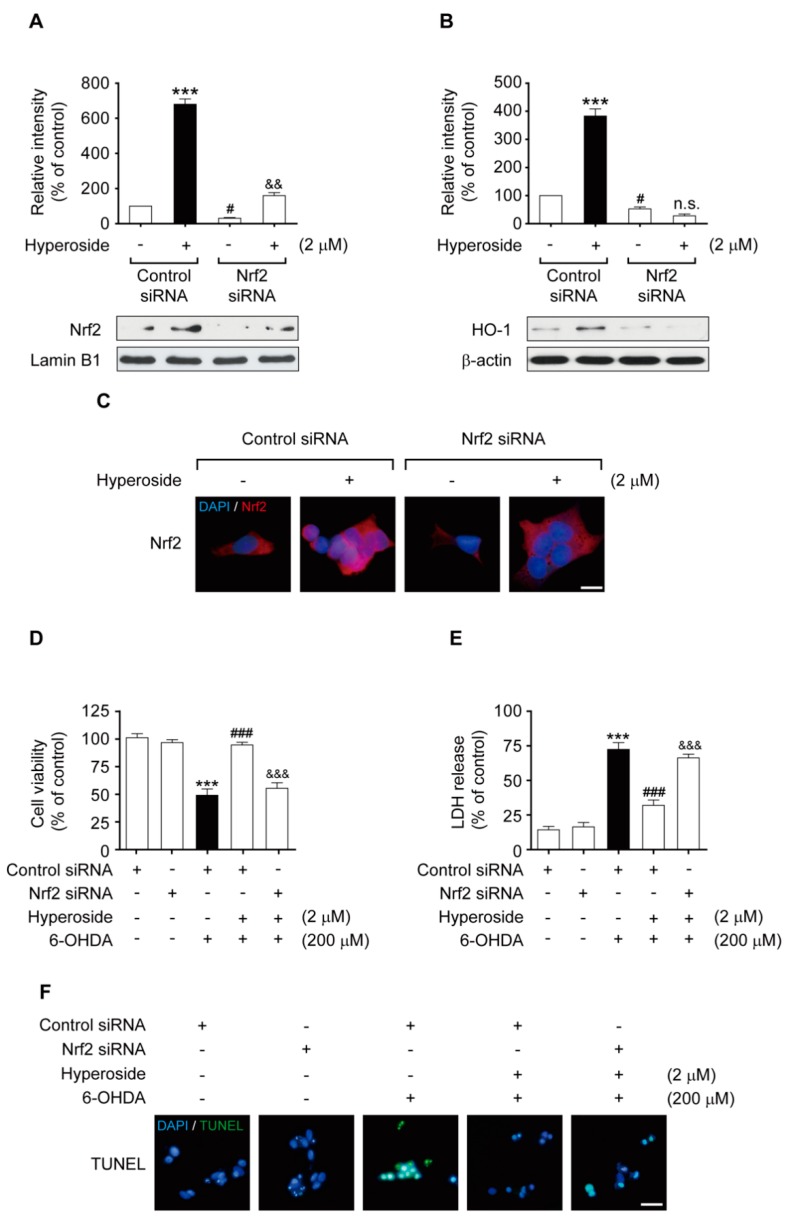
Nrf2 gene knockdown eliminated the neuroprotective effects of hyperoside on Nrf2-mediated HO-1 transcriptional induction. Cells were transiently transfected with an Nrf2 siRNA, as described in the Material and Methods, and then treated with or without 2 μM hyperoside for 4 h prior to exposed to 200 μM 6-OHDA for 24 h. Levels of Nrf2 (**A**), HO-1 (**B**), and β-actin were evaluated by Western blot analysis. Error bars indicate the mean ± SEM (*n = 3*). *** *p* < 0.001 vs. the (−) control siRNA group. ^#^ *p* < 0.001 vs. (+) control siRNA group. ^&&^ *p* < 0.01 vs. the (+) Nrf2 siRNA group. Knockdown of Nrf2 abolished the neuroprotective effects of hyperoside on cell death (**D**) and LDH release (**E**) induced by 6-OHDA. Cells were transiently transfected with the siRNA Nrf2 plasmid construct and then treated with 2 μM hyperoside for 4 h, followed by exposure to 200 μM 6-OHDA for 24 h. Cells were pretreated with the indicated concentrations of hyperoside for 4 h. Nrf2 was detected using an anti-Nrf2 antibody and a Texas red^®^-conjugated secondary antibody (**C**, 100× magnification, scale bar: 50 μm). DNA fragmentation was assayed using TUNEL fluorescent dye (**F**). Nuclei were stained with Hoechst 33258. Images shown are representative of three experiments and were visualized by fluorescence microscopy. Error bars indicate the mean ± SEM (*n = 6*). ***p < 0.001 vs. the control group. ^###^ *p* < 0.001 vs. the 6-OHDA-treated group. ^&&^ *p* < 0.01 and ^&&&^ *p* < 0.001 vs. the 6-OHDA plus sieboldin-treated group. Scale bar: 100 μm.

To clarify whether the neuroprotective effects of hyperoside are accompanied by Nrf2 activation, we performed transient Nrf2-siRNA transfection. Activation of Nrf2 and HO-1 expression following hyperoside treatment was significantly eliminated by siRNA-mediated Nrf2 knockdown (Figure 6A,B, *p* < 0.05, *p* < 0.01 and *p* < 0.001, respectively). The immunostaining assay revealed the nuclear accumulation of Nrf2 in cells treated with hyperoside (**5**); however, its translocation from the cytosol to the nucleus and its subsequent activation was significantly abolished by knockdown of the Nrf2 gene using siRNA system (Figure 6C). Similarly, the neuroprotective effects of hyperoside (**5**) against 6-OHDA-induced cytotoxicity were abrogated upon transfection of the cells with Nrf2-siRNA, suggesting that the neuroprotective effects of hyperoside are mediated via Nrf2 activation (Figure 6C–E, *p* < 0.001, respectively). As seen in the representative images, TUNEL staining revealed significant DNA fragmentation in the nucleus after exposure to 6-OHDA, whereas transient transfection with Nrf2-siRNA considerably abolished the nuclear translocation and activation of Nrf2 in cells (Figure 6F).

## 3. Discussion

Here we evaluated the neuroprotective effects of hyperoside, the most potent active compound isolated from *A. tegmentosum*, on 6-OHDA-induced oxidative stress, as an in vitro cellular PD model, using human dopaminergic SH-SY5Y cells. We used 6-OHDA to induce oxidative stress, and subsequently, cell death in these dopaminergic neurons. Our findings indicate that hyperoside inhibits 6-OHDA-induced neurotoxicity, most likely by preventing oxidative stress and activating Nrf2-mediated HO-1 signaling.

Hyperoside (quercetin 3-*O*-galactoside), a phenolic compound present in the bark of *A. tegmentosum*, has displayed multiple biological protective effects against chronic diseases through its antioxidant [47], anti-inflammatory [48] and anticancer [49] activities; moreover, it effectively prevents amyloid beta-induced neurotoxicity [50], oxygen-glucose deprivation-reperfusion-induced cortical neuron injury [51] and chronic stress-exposed depressive-like behavior [52] in vivo. There is growing evidence of the effects of hyperoside on neurodegenerative diseases; however, the mechanisms underlying these effects of hyperoside in PD have not yet been addressed. Moreover, there have been no direct studies demonstrating the molecular mechanisms underlying the antioxidant activities of hyperoside in 6-OHDA-induced neurotoxicity in human dopaminergic SH-SY5Y cells. To our knowledge, this is the first report to demonstrate the neuroprotective effect of hyperoside against 6-OHDA-induced oxidative damage in human dopaminergic SH-SY5Y cells via the Nrf2-mediated HO-1 signaling pathway in a cellular PD model.

As previously mentioned, PD is a progressive condition characterized by selective dopaminergic neuron loss in the substantia nigra pars compacta (SNpc) [53]. Although the etiology and pathology of PD are not entirely understood, many studies have suggested that, in its pathogenesis, oxidative stress plays an essential role in the loss of dopaminergic neurons [7]. It is generally recognized that oxidative stress from excessive accumulation of ROS can injure neuronal cells, as well as trigger mitochondrial deterioration and apoptotic cascade events [54,55]. In particular, SNpc is considerably more vulnerable to oxidative stress, as it contains inherently fewer antioxidant enzymes than other brain regions [56]. Moreover, PD is primarily caused by ROS production induced by auto-oxidation and mitochondrial membrane dysfunction in dopaminergic neurons [57]. Thus, it is essential to improve and enhance the antioxidant defense system to control oxidative stress at the cellular level.

There is a large body of evidence suggesting that oxidative stress is a major contributor to mitochondrial-mediated apoptotic neuronal cell death, and that the preventive effects of antioxidants on these causative cellular events can effectively block cell death [6]. Besides, abnormal intracellular ROS accumulation is associated with PD, and ultimately, leads to dopaminergic neuronal cell death. Many studies have demonstrated that neuronal injury is an essential source of ROS in the brain; thus, the suppression of excessive ROS production might be an alternative strategy to protect neurons [58]. Conversely, mitochondrial membrane dysfunction is well established as an associated event in many forms of apoptotic and necrotic cell death, with evidence showing that both modes of cell death may play a role in PD [59]. Mitochondrial dysfunction may result in apoptotic cell death via a reduction in the mitochondrial membrane potential (MMP), leading to an apoptotic signaling pathway cascade [60]. Therefore, we tested the neuroprotective effect of hyperoside on 6-OHDA-induced excess ROS production and mitochondrial membrane dysfunction. 

In this study, we found that the loss of mitochondrial metabolic activity and membrane integrity in response to 6-OHDA was significantly prevented by pretreatment with hyperoside, compared to NAC (10 mM); moreover, even 0.5–2 μM hyperoside was sufficient to exhibit a potent antioxidant effect that benefits clinical drug development. Furthermore, we found that hyperoside significantly suppressed intracellular ROS accumulation and mitochondrial membrane dysfunction in SH-SY5Y cells, which is in agreement with previous observations by other groups who reported it to be an effective protective agent against oxidative stress-mediated cell death [47,61]. Based upon our findings, we aimed to elucidate the underlying molecular mechanisms responsible for the neuroprotective effects of hyperoside in the cellular PD model.

The transcription factor Nrf2 plays a key role in the adaptive response to oxidative stress by exerting neuroprotective effects via the induction of a series of phase II detoxification enzymes. Of late, specific Nrf2 inducers have been studied clinically, and Nrf2 has been considered an emerging therapeutic target [62]. Despite the neuroprotective abilities of compounds with Nrf2 activity, their use in PD is uncertain due to a lack of information regarding their ability to pass through the central nervous system. Thus, in the search for compounds that activate Nrf2 signaling in the brain, those that quickly pass through the blood–brain barrier and can be taken up by neuronal cells representing potential drug targets [63,64]. Therefore, we hypothesized that the neuroprotective actions of hyperoside were affected by the activated Nrf2-mediated induction of the HO-1 transcriptional signaling pathway. To better define the neuroprotective role of hyperoside, we examined its effects on the nuclear translocation of Nrf2, as well as the activation of its downstream signaling HO-1, a key protein transcriptional regulated by Nrf2. We found that hyperoside activated Nrf2 nuclear translocation in neuronal cells, demonstrating activation of neuroprotective pathways. Our finding suggests that nuclear translocation of Nrf2 and Nrf2-mediated ARE activation has a pivotal role in the increase in HO-1 induction by hyperoside. Nrf2 activation leads to the induction of HO-1 transcription, which, consequently, results in potent anti-inflammatory and antioxidant effects [65,66]. A large body of evidence suggests that HO-1, a critical neuroprotective enzyme, plays a crucial role in maintaining antioxidant homeostasis during cellular stress [67]. The induction of HO-1 provides neuroprotection; thus, elevating the HO-1 expression by using a pharmacologic modulator may represent a valid strategy for therapeutic intervention [68]. To obtain direct evidence of the role of Nrf2-mediated HO-1 signaling in hyperoside-induced neuroprotective activities, we examined the effects of Nrf2-siRNA transfection on hyperoside neuroprotection using multiple analyses of human dopaminergic SH-SY5Y cells. Our study demonstrated that hyperoside induces HO-1 expression levels and that Nrf2 knockdown by small interfering RNA (siRNA) blocked hyperoside-induced neuroprotective effects, indicating that Nrf2/HO-1 plays an essential role in mediating the neuroprotective effects of hyperoside. This study validates the neuroprotective effect of hyperoside against 6-OHDA-induced oxidative stress and neurotoxicity via the alleviation of intracellular ROS accumulation, mitochondrial dysfunction and eventually, apoptotic cell death. Collectively, the results of this study demonstrate that hyperoside exhibits remarkable neuroprotective effects against 6-OHDA-induced oxidative stress and neurotoxicity by activating the Nrf2-mediated HO-1 transcriptional signaling pathway.

## 4. Materials and Methods

### 4.1. Cell Culture and Treatment

Human dopaminergic neuroblastoma SH-SY5Y cells were obtained from the American Tissue Culture Collection (ATCC^®^ CRL-2142, Manassas, VA, USA). Cells were maintained in Dulbecco′s Modified Eagle Medium (DMEM) supplemented with 10% heat-inactivated fetal bovine serum (FBS) (*v*/*v*) and 0.1% penicillin/streptomycin at 37 °C in an atmosphere of 5% CO_2_ and 95% air. Hyperoside and *N*-acetylcysteine (NAC) were dissolved in dimethyl sulfoxide (DMSO); the stock solution was added directly to the culture medium. 6-hydroxydopamine (6-OHDA) was prepared as a 20 mM stock solution immediately prior to use, and was diluted in PBS to the indicated final concentration. The final solvent concentration was always < 0.1% (*v*/*v*). No significant cytotoxicity was observed in any of the experiments. 

### 4.2. Measurement of Cell Viability

Cells (2.5 × 10^5^ cells/well in 24-well plates) were incubated at 37 °C with 200 μM 6-OHDA for 24 h with or without hyperoside (**5**) or *N*-acetylcysteine (NAC) pretreatment, and then treated with 3-(4,5-dimethyl thiazol-2-yl)-2,5-diphenyl tetrazolium bromide (MTT) solution (5 mg/mL) for 4 h. The dark-blue formazan crystals formed in viable cells were dissolved in DMSO; subsequently, the absorbance of each reaction product was measured using a microplate reader (SpectraMax 250, Molecular Devices, Sunnyvale, CA, USA) at 540 nm.

### 4.3. Measurement of Lactate Dehydrogenase (LDH) Release

Extracellular and intracellular Lactate Dehydrogenase (LDH) activities were spectrophoto-metrically measured as cytotoxicity using an LDH cytotoxicity assay kit (Thermo Fisher Scientific Inc., Bartlesville, OK, USA), according to the manufacturer′s protocol. Cells (2.5 × 10^5^ cells/well in 24-well plates) were incubated at 37 °C with 200 μM 6-OHDA for 24 h with or without hyperoside or NAC pretreatment; subsequently, the supernatant was assayed by adding 100 μL of the reaction mixture to each well at room temperature. The absorbance of each sample was read at 490 nm using a microplate reader.

### 4.4. Terminal Deoxynucleotidyl Transferase-Mediated dUTP-Biotin Nick-End Labeling (TUNEL) Assay

Terminal deoxynucleotidyl transferase dUTP nick end labeling (TUNEL) staining was detected using the DeadEnd™ Fluorometric TUNEL System (Promega, Madison, WI, USA), according to the manufacturer′s protocol. Briefly, cells (5 × 10^5^ cells/well in 6-well plates) were plated on coverslips coated with poly-d-lysine for 24 h. Then, cells were incubated at 37 °C with 200 μM 6-OHDA for an additional 24 h, with or without hyperoside pretreatment. Finally, the cells were washed with phosphate-buffered Saline (PBS). For the immunocytochemistry assay, cells were immersed in 4% paraformaldehyde for 15 min, washed twice with PBS, and finally, permeabilized with 0.2% Triton X-100 for 5 min. Each glass coverslip was covered with equilibration buffer for 10 min. Then, the buffer was aspirated, and the glass coverslips were incubated with terminal deoxynucleotidyl transferase (TdT) buffer for 1 h at 37 °C. Following incubation, the cells were washed again with PBS and observed under a fluorescence microscope (20×). TUNEL positive cells were quantified by counting the degree of DNA fragmentation in different entire areas of the slide in three random fields of each group.

### 4.5. Measurement of Intracellular Reactive Oxygen Species (ROS) Accumulation

Cells (2.5 × 10^5^ cells/well in 48-well plates or 2 × 10^5^ cells in 4-well chamber slides) were incubated at 37 °C with 200 μM 6-OHDA for 4 h with or without hyperoside pretreatment. The cells were washed with PBS and incubated with 10 mM 2′,7′-dichlorodihydrofluorescein diacetate (DCFH-DA) in the dark. After 30 min incubation at 37 °C, cells were examined using a fluorescence spectrophotometer (SpectraMax 250, Molecular Devices) at an excitation wavelength of 530 nm and an emission wavelength of 480 nm. DCFH-DA fluorescence representative images were examined by fluorescence microscopy (20×) (BX51, Olympus Optical Co., Ltd., Tokyo, Japan).

### 4.6. Measurement of Intracellular Mitochondrial Membrane Potential (MMP)

Cells (2.5 × 10^5^ cells/well in 48-well plates or 2 × 10^5^ cells in 4-well chamber slides) were incubated at 37 °C with 200 μM 6-OHDA for 24 h with or without hyperoside pretreatment. The cells were washed with PBS, and 10 μM rhodamine 123 was added. After 30 min incubation at 37 °C, the cells were examined at 530 nm using a fluorescence spectrophotometer at an excitation wavelength of 480 nm [69]. Rhodamine123 fluorescence representative images were examined under a fluorescence microscope (20×).

### 4.7. RNA Isolation and Reverse Transcription-Polymerase Chain Reaction (RT-PCR)

Cells (1 × 106 cells/well in 6-well plates) were incubated at 37 °C with hyperoside for 0, 1, 2, 4, 6 and 12 h. Total mRNA was isolated using Trizol^®^ reagent (Invitrogen, Carlsbad, CA, USA) and reverse-transcription was performed using the Superscript^®^-III kit (Invitrogen), according to the manufacturers′ instructions. Amplification of cDNA was carried out using specific primers: HO-1 (forward, 5’- CCAGAAGAGCT GCACCGCAA-3’ and reverse, 5’-GCTGGATGTTGAGCAGGAAC-3’) and glyceraldehyde 3-phosphate dehydrogenase (GAPDH, forward, 5’- ACCACAGTCCATGCCATCAC-3’ and reverse, 5’-TCCACCACCCTGTTGCTGTA-3’). The amplified polymerase chain reaction (PCR) products were separated by staining with ethidium bromide (EtBr) on 1.5% agarose gel electrophoresis in Tris borate/EDTA buffer (890 mM Tris-Base, 890 mM boric acid, 20 mM EDTA, pH 8.3, alkali) for 30 min at 100 V. Specific bands were compared after visualization and imaging using a UV transilluminator. The mRNA bands were quantified by densitometric analysis using ImageJ software (NIH Image in the public domain, NIH, Bethesda, MD, USA).

### 4.8. Transient Transfection with siRNA

Transfection of cells with siRNAs was performed using the Lipofectamine^®^ 2000 transfection reagent (invitrogen) according to the manufacturer′s instructions. Commercially-available human Nrf2-specific small interfering RNA (siRNA) (Santa Cruz Biotechnology, Santa Cruz, CA, USA) and negative control siRNAs (Santa Cruz Biotechnology, Santa Cruz, CA, USA) were used for transfection. To knockdown Nrf2 expression, Lipofectamine^®^ 2000 transfection reagent (10 μL) was added to 200 μL of serum-free Opti-MEM^®^, containing 10 μM each siRNA, followed by 20 min incubation at room temperature. Gene silencing was performed for 24 h; subsequently, transfected cells were treated with 2 μM hyperoside for 4 h in the presence or absence of 200 μM 6-OHDA for 24 h. Cell viability, LDH, TUNEL assays, and Western blot analysis were performed as described in Section 4.2, Section 4.3, Section 4.4, and Section 4.10, respectively.

### 4.9. Cytosolic and Nuclear Lysate Preparation

Nuclear and cytosolic fractions were prepared with a commercial kit according to the manufacturer′s instructions (Thermo Scientific Inc., Rockford, IL, USA). All steps were carried out on ice, unless stated otherwise. Protease and phosphatase inhibitor cocktail tablets (Complete Mini and Phospho STOP, Roche Applied Science, Indianapolis, IN, USA, respectively) were added to each buffer just prior to use. Briefly, cells (5 × 10^6^ cells/well in 100 mm^2^ cell culture dishes) were treated with hyperoside for 0, 1, 2, 4, 6 or 12 h at 37 °C. Protein concentrations in the samples were determined using a bicinchoninic acid assay (BCA assay) protein assay kit (Thermo Scientific Inc., Rockford, IL, USA). Nrf2 levels were assessed using western blot analysis, as described below.

### 4.10. Western Blot Analysis

Cells (1 × 10^6^ cells/well in 6-well plates) were plated and treated with hyperoside for 0, 1, 2, 4, 6 or 12 h; then the cells were washed and collected with PBS and centrifuged at 400× *g* for 5 min. The cell pellets were extracted using the T-per tissue protein extraction buffer (Thermo Fisher Scientific Inc., Bartlesville, OK, USA), containing protease and phosphatase inhibitor cocktail tablets (Complete Mini and Phospho STOP, respectively). After centrifugation at 10,000× *g* for 15 min, the supernatant was separated and stored at −70 °C. The protein concentration in the samples was determined using a BCA protein assay kit. Protein samples were separated on an 8–12% SDS–polyacrylamide gel and transferred to a polyvinylidene difluoride membrane (Bio-Rad Laboratories, Inc., Hercules, CA, USA) that was further blocked with 5% skim milk, containing 0.5 mM Tris-HCl (pH 7.5), 150 mM NaCl and 0.1% Tween-20 for 1 h at room temperature. After blocking for 1 h at room temperature, the membrane was reacted with primary antibodies overnight at 4 °C (each antibody at a dilution of 1:1000; HO-1, laminin B1 and Nrf2, except for β-actin (1:20,000)). After three washes with tris-buffered saline combined with 0.1% polysorbate 20 (Tween-20) (this combination referred to as TBST or TTBS), the blots were incubated with horseradish-peroxidase-conjugated anti-rabbit or anti-mouse secondary antibodies in TBST at a 1:5000 dilution for 1 h at room temperature. Then the blots were washed three times in this TBST buffer. The blots were developed using an enhanced chemiluminescence detection method in a mixture of SuperSignal^®^ West Pico Chemiluminescent Substrate reagents (Thermo Fisher Scientific Inc., Bartlesville, OK, USA). Protein bands were quantified via densitometric analysis using ImageJ software from NIH (Bethesda, MD, USA).

### 4.11. Transient Transfection and Dual-Luciferase Assay

Luciferase activity was assayed using a dual-luciferase assay kit (Promega, Madison, WI, USA), according to the manufacturer′s instructions. To determine promotor activity, antioxidant response element (ARE) reporter constructs were purchased from SABiosiences (Qiagen, Valencia, CA, USA). Brief, cells were plated in 24-well plates overnight. After overnight transfection, the cells were exposed to hyperoside for indicated times and concentrations. Luminescence in cell lysates was measured using a single tube luminometer (FB12, Berthold Detection Systems GmbH, Pforzheim, Germany).

### 4.12. Immunocytochemistry

SH-SY5Y cells (2 × 10^5^ cells/well) were grown on poly-d-lysine coated chamber slides overnight and treated with hyperoside. After washing with PBS, cells were fixed with 4% paraformaldehyde for 15 min and permeabilized with 0.1% Triton X-100 in PBS for 10 min. After washing with PBS, cells were blocked in 5% BSA solution in PBS for 30 min and incubated overnight with anti-Nrf2 (1:250) at 4 °C. Subsequently, the cells were washed with PBS and reacted with Texas red^®^-conjugated goat anti-mouse IgG antibodies (1:250) and Hoechst 33258 (5 μg/mL) for 1 h. After washing with PBS, the cells were mounted on glass slides in Permafluor aqueous mounting fluid. The Nrf2 nuclear translocation was observed under a fluorescence microscope (20×); the results are representative of three independent experiments.

### 4.13. Statistical Analysis

Data were analyzed with Prism 5.0 software (GraphPad Software, Inc., San Diego, CA, USA) and are presented as the means ± the standard error of the mean (SEM). Statistical analyses were performed using a one way analysis of variance (ANOVA), followed by the Newman-Keuls test. Any *p* values < 0.05 were considered statistically significant.

## 5. Conclusions

Our study demonstrates that compounds **1**, **2**, **3**, **5**, **6**, **7**, **9**, **11** and **13** isolated from *A. tegmentosum* showed neuroprotective effects on human dopaminergic neuron cells. Among these active compounds, hyperoside (**5**) showed the most potent neuroprotective effect against 6-OHDA-induced cell toxicity. In terms of its molecular mechanism, hyperoside significantly inhibited oxidative stress-mediated neuronal cell toxicity induced by 6-OHDA. We also demonstrated that hyperoside induces the activation of Nrf2-dependent cellular antioxidant defenses, leading to the inhibition of 6-OHDA-induced oxidative injuries. Importantly, this is the first report, to our knowledge, demonstrating that Nrf2/HO-1 signaling may contribute to the neuroprotection mediated by hyperoside against 6-OHDA-induced oxidative stress. 

Our findings provide direct experimental evidence and a molecular interpretation for the antioxidant properties of hyperoside in neurodegenerative diseases and suggest that *A. tegmentosum* and its constituents have the potential for development as neuroprotective agents for the prevention of PD progression and related diseases.

## Supplementary Materials

Supplementary materials can be found at https://www.mdpi.com/1422-0067/20/23/5832/s1. General experimental procedures, plant materials, isolation and purification, chemicals and reagents, neuroprotective effects of the isolated compounds (Figure S1) and NMR data of the compounds are available free of charge on the internet.
