# Supplementary material for: Suppression of 6-Hydroxydopamine-Induced Oxidative Stress by Hyperoside Via Activation of Nrf2/HO-1 Signaling in Dopaminergic Neurons
Source: Int J Mol Sci. 2019 Nov 20;20(23):5832. doi: 10.3390/ijms20235832 (PMC6929192; doi:10.3390/ijms20235832)

Supplementary Materials

Suppression of 6-Hydroxydopamine-induced Oxidative Stress by Hyperoside via Activation of Nrf2/HO-1 Signaling in Dopaminergic Neurons

Seung-Hwan Kwon [1,2], Seoung Rak Lee [3], Yong Joo Park [3], Moonjin Ra [4], Yongjun Lee [4], Changhyun Pang [5,6,*] and Ki Hyun Kim [3,*]

[1] Neuroregeneration and Stem Cell Programs, Institute for Cell Engineering, The Johns Hopkins University School of Medicine, Baltimore, MD 21205, USA.; skwon28@jhmi.edu

[2] Department of Neurology, The Johns Hopkins University School of Medicine, Baltimore, MD 21205, USA

[3] School of Pharmacy, Sungkyunkwan University, Suwon 16419, Korea; davidseoungrak@gmail.com (S.R.L); pyj084@msn.com (Y.J.P.)

[4] Hongcheon Institute of Medicinal Herb, 101 Yeonbongri, Hongcheon 25142, Korea; ramj90@himh.re.kr (M.R.); leerayongjun@gmail.com (Y.L.)

[5] School of Chemical Engineering, Sungkyunkwan University, Suwon 16419, Korea

[6] SKKU Advanced Institute of Nanotechnology Sungkyunkwan University, Suwon 16419, Korea

* Correspondence: chpang@skku.edu (C.P.); khkim83@skku.edu (K.H.K.); Tel.: +82-31-290-7341 (C.P.); +82-31-290-7700(K.H.K.)

Materials and Methods

1. General experimental procedures

Optical rotations were calculated using a Jasco P-1020 polarimeter (Jasco, Easton, MD, USA). Electrospray ionization (ESI) and HR-ESI mass spectra were transcribed using a Waters Micromass Q-TofUltima ESI-TOF mass spectrometer (Waters, New York, NY, USA). NMR spectra were recorded on a Varian UNITY INOVA 700 NMR spectrometer operating at 700 MHz (^{1}H) and 175 MHz (^{13}C), with chemical shifts given in ppm (δ). Preparative high-performance liquid chromatography (HPLC) was performed on a Waters 1525 Binary HPLC pump with Waters 996 Photodiode Array Detector (Waters Corporation, Milford, CT, USA). Semi-preparative HPLC utilized a Shimadzu Prominence HPLC System with SPD-20A/20AV Series Prominence HPLC UV-Vis Detectors (Shimadzu, Tokyo, Japan). LC/MS analysis was conducted on an Agilent 1200 Series HPLC system (Agilent Technologies, Santa Clara, CA, USA) equipped with a diode array detector and a 6130 Series ESI mass spectrometer using an analytical Kinetex (4.6 × 100 mm, 3.5 μm). Silica gel 60 (Merck, 70-230 mesh and 230-400 mesh) and RP-C_{18} silica gel (Merck, 40-63 μm) were used for column chromatography. The packing material for molecular sieve column chromatography was Sephadex LH-20 (Pharmacia, Uppsala, Sweden). Precoated silica gel F_{254} plates and RP-18 F_{254s} plates (Merck, Darmstadt, Germany) were used for TLC. Spots were detected on TLC under UV light or by heating after spraying with anisaldehyde-sulfuric acid.

2. Plant Materials

Bark samples of *A. tegmentosum* were obtained from Hongcheon and Jeongseon in Gangwon province, Korea, in June 2013. The material was confirmed by one of the authors (M. Ra). A voucher specimen (MIMH-35) was deposited in the herbarium of Hongcheon Institute of Medicinal Herb, Hongcheon, Korea.

3. Isolation and Purification

The bark of *A. tegmentosum* was dried at 60°C for 24 h and was pulverized to obtain 200 g of dried and pulverized *A. tegmentosum* bark. The material was extracted with distilled water (1 L) at 90°C for 10 h and then filtered. The filtrate was concentrated *in vacuo* to obtain

the resultant extracts (13.2 g), which were suspended in distilled water (700 mL × 3) and successively solvent partitioned with hexane, CH_2Cl_2, EtOAc, and *n*-BuOH to yield 62.1 g, 32.2 g, 22.4 g, and 37.3 g, respectively. The EtOAc soluble fraction was loaded onto a Diaion HP-20 chromatography column and was fractionated with 500 mL of each solvent system of 20%, 40%, 60%, 80%, and 100% MeOH in H_2O. Based on TLC analysis, the 80% and 100% MeOH miscible fractions were combined into one fraction (4.2 g), separated by RP-C_{18} silica gel (230-400 mesh) column chromatography, and eluted with a gradient solvent system of MeOH-H_2O (1:1-1:0, v/v) to obtain three fractions (A-C). Fraction B (3.3 g) was subjected to silica gel (230-400 mesh) column chromatography and separated with a gradient solvent system of EtOAc-MeOH (30:1-1:1, v/v) to yield three fractions (BA-BC). Further, three fractions (BA1-3) were acquired from fraction BA (511 mg) by Sephadex LH-20 column chromatography eluted with 100% MeOH. Fraction BA1 (147 mg) was further fractionated by silica gel (70-230 mesh) column chromatography with a gradient solvent system of CH_2Cl_2-MeOH (50:1 to 1:1, v/v) to provide three subfractions (BA11-16). Subfraction BA12 (51 mg) was further purified by semipreparative reverse-phase HPLC eluted with 37% MeOH/H_2O (flow rate: 2 mL/min) to obtain compound **2** (3.0 mg, t_R = 50.0 min). Fraction BA2 (187 mg) was loaded onto a silica gel (70-230 mesh) chromatography column and fractionated with a gradient solvent system of EtOAc-MeOH (50:1 to 5:1, v/v) to yield three subfractions (BA21-23). Compounds **10** (7.6 mg, t_R = 58.0 min), **12** (1.0 mg, t_R = 20.0 min), **13** (7.3 mg, t_R = 26.0 min), and **14** (4.6 mg, t_R = 55.0 min) were obtained from subfraction BA22 (62 mg) using semipreparative reverse-phase HPLC with an isocratic solvent system of 28% MeOH (flow rate: 2 mL/min). Five fractions (BB1-5) were acquired from fraction BB (564 mg) by Sephadex LH-20 column chromatography eluted with 100% MeOH. Fraction BB4 (136 mg) was further fractionated using preparative reverse-phase HPLC with a gradient solvent system of MeOH-H_2O (3:7-4:6, flow rate: 5 mL/min) to yield four subfractions (BB41-44). Subfraction BB41 (15 mg) was then purified by semipreparative reverse-phase HPLC with an isocratic solvent system of 30% MeOH (flow rate: 2 mL/min) to isolate compound **15** (8.1 mg, t_R = 33.0 min). Compound **1** (3.5 mg, t_R = 40.0 min) was isolated from subfraction BB42 (8 mg) by semipreparative reverse-phase HPLC with an isocratic solvent system of 33% MeOH (flow rate: 2 mL/min). Subfraction BB44 (12 mg) was purified by semipreparative reverse-phase HPLC eluted with an isocratic solvent system of 36% MeOH (flow rate: 2 mL/min) to obtain compound **11** (3.9 mg, t_R = 50.0 min). Fraction BB5 (84 mg) was purified using semipreparative reverse-phase HPLC eluted with an isocratic solvent

system of 32% MeOH (flow rate: 2 mL/min) to provide compound **9** (4.0 mg, t_R = 42.0 min). Fraction BC (928 mg) was subjected to Sephadex LH-20 column chromatography and was separated with 100% MeOH to give eight subfractions (BC1-8). Compound **3** (3.8 mg, t_R = 20.0 min) was obtained from subfraction BC5 (43 mg) by semipreparative reverse-phase HPLC eluted with an isocratic solvent system of 38% MeOH (flow rate: 2 mL/min). Subfraction BC6 (9 mg) was purified by semipreparative reverse-phase HPLC eluted with an isocratic solvent system of 44% MeOH (flow rate: 2 mL/min) to obtain compound **4** (1.7 mg, t_R = 40.0 min). Subfraction BC7 (22 mg) was also purified by semipreparative reverse-phase HPLC with an isocratic solvent system of 44% MeOH (flow rate: 2 mL/min) to acquire compounds **5** (1.0 mg, t_R = 40.0 min), **6** (1.2 mg, t_R = 44.0 min), **7** (1.5 mg, t_R = 47.0 min), and **8** (3.9 mg, t_R = 50.0 min).

4. Chemicals and reagents

N-acetylcysteine (NAC), 2,7’-dichlorofluorescein diacetate (DCFH-DA), dimethyl sulfoxide (DMSO), 3-(4,5-dimethyl thiazol-2-yl)-2,5-diphenyl tetrazolium bromide (MTT), 6-hydroxydopamine (6-OHDA), Hoechst 33258, rhodamine 123, and anti-β-actin antibody were purchased from Sigma Chemicals (St. Louis, MO, USA). Dulbecco’s modified Eagle’s medium (DMEM) was obtained from Hyclone (Logan, UT, USA). Fetal bovine serum (FBS), 0.25% trypsin-EDTA, L-glutamine, and a penicillin/streptomycin mixture were obtained from GIBCO–BRL (Grand Island, NY, USA). Rabbit anti-Nrf2 and rabbit anti-HO-1 antibodies were purchased from Abcam Inc. (Cambridge, MA, USA). Anti-rabbit horseradish peroxidase-linked IgG antibodies were purchased from Jackson ImmunoResearch Inc. (West Grove, PA, USA). Texas red®-conjugated goat anti-rabbit IgG antibody, Opti-MEM®, and Lipofectamine® 2000 transfection reagents were purchased from Life Technologies Co., (Invitrogen, Eugene, OR, USA). Human-specific Nrf2-small interfering RNA (siRNA, cat. sc-37030) and control siRNA (cat. sc-37007) constructs were obtained from Santa Cruz Biotechnology Inc. (Santa Cruz, CA, USA). PCR primers were synthesized by Cosmogenetech (Seoul, Republic of Korea). All other chemicals were purchased from Sigma Chemical Company and were of analytical grade.

5. Measurement of cell viability

Cells (2.5×10^5 cells/well in 24-well plates) were incubated at 37°C with 200 μM 6-OHDA for 24 h with or without isolated compounds **1**–**15** or NAC pretreatment and, then,

treated with MTT solution (5 mg/mL) for 4 h. The dark-blue formazan crystals formed in viable cells were dissolved in DMSO; subsequently, the absorbance of each reaction product was measured using a microplate reader (SpectraMax 250, Molecular Devices, Sunnyvale, CA, USA) at 540 nm.

6. Statistical analysis

All data were analyzed with Prism 5.0 software (GraphPad Software, Inc., San Diego, CA, USA) and are presented as the means ± S.E.M. Statistical analyses were performed using a one-way analysis of variance (ANOVA), followed by the Newman-Keuls test. IC_{50} values were calculated to Nonlinear regression curve fit, followed by the Sigmodal dose-response (variable slope). p values < 0.05 were considered statistically significant.

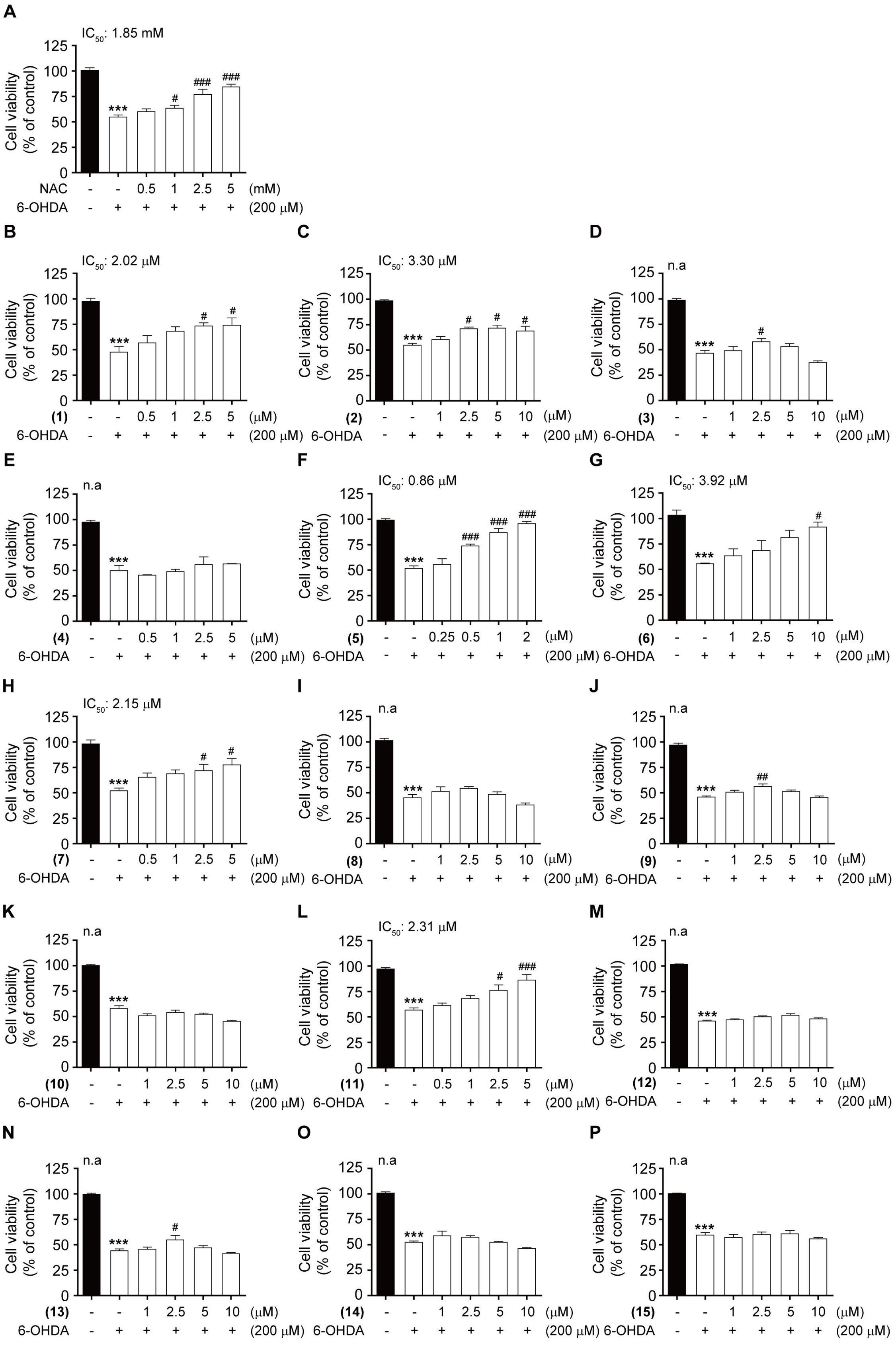
A
IC50: 1.85 mM
Cell viability (% of control)
125 100 75 50 25 0
***
#
###
###
NAC - - 0.5 1 2.5 5 (mM)
6-OHDA - + + + + + (200 μM)
B
IC50: 2.02 μM
***
#
#
(1) - - 0.5 1 2.5 5 (μM)
6-OHDA - + + + + + (200 μM)
C
IC50: 3.30 μM
***
#
#
#
(2) - - 1 2.5 5 10 (μM)
6-OHDA - + + + + + (200 μM)
D
n.a
***
#
(3) - - 1 2.5 5 10 (μM)
6-OHDA - + + + + + (200 μM)
E
n.a
***
(4) - - 0.5 1 2.5 5 (μM)
6-OHDA - + + + + + (200 μM)
F
IC50: 0.86 μM
***
###
###
###
(5) - - 0.25 0.5 1 2 (μM)
6-OHDA - + + + + + (200 μM)
G
IC50: 3.92 μM
***
#
(6) - - 1 2.5 5 10 (μM)
6-OHDA - + + + + + (200 μM)
H
IC50: 2.15 μM
***
#
#
(7) - - 0.5 1 2.5 5 (μM)
6-OHDA - + + + + + (200 μM)
I
n.a
***
(8) - - 1 2.5 5 10 (μM)
6-OHDA - + + + + + (200 μM)
J
n.a
***
##
(9) - - 1 2.5 5 10 (μM)
6-OHDA - + + + + + (200 μM)
K
n.a
***
(10) - - 1 2.5 5 10 (μM)
6-OHDA - + + + + + (200 μM)
L
IC50: 2.31 μM
***
#
###
(11) - - 0.5 1 2.5 5 (μM)
6-OHDA - + + + + + (200 μM)
M
n.a
***
(12) - - 1 2.5 5 10 (μM)
6-OHDA - + + + + + (200 μM)
N
n.a
***
#
(13) - - 1 2.5 5 10 (μM)
6-OHDA - + + + + + (200 μM)
O
n.a
***
(14) - - 1 2.5 5 10 (μM)
6-OHDA - + + + + + (200 μM)
P
n.a
***
(15) - - 1 2.5 5 10 (μM)
6-OHDA - + + + + + (200 μM)

Figure S1. Neuroprotective effects of the isolated compounds on 6-OHDA-induced loss of cell viability in SH-SY5Y cells. Cells were pretreated with the indicated concentrations of the test compounds or NAC for 4 h and, then, treated with 200 μM 6-OHDA for 24 h. Error bars indicate the mean ± S.E.M. (*n* = *6*). IC_{50} values were calculated to Nonlinear regression curve fit, followed by the Sigmodal dose-response (variable slope). *** $p < 0.001$ *vs. the* control group. # $p < 0.05$, ## $p < 0.01$, and ### $p < 0.001$ *vs. the* 6-OHDA-treated group. NAC: *N*-acetylcysteine.

NMR data of compounds 1-15

*1. The ^{1}H NMR spectrum of **1***

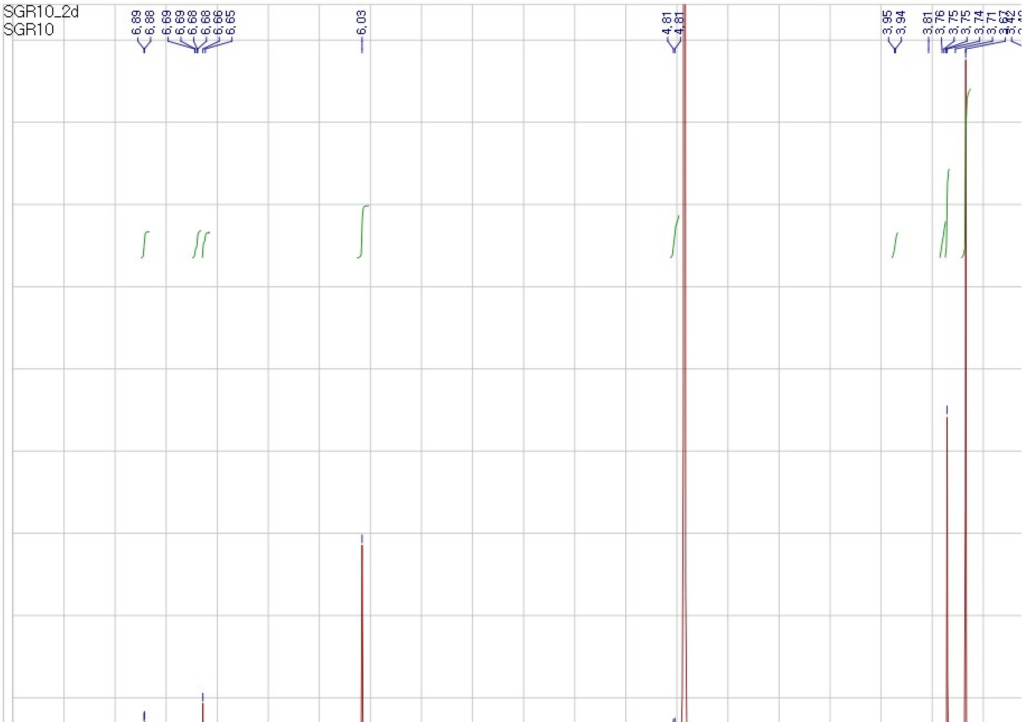

*2. The ^{13}C NMR spectrum of **1***

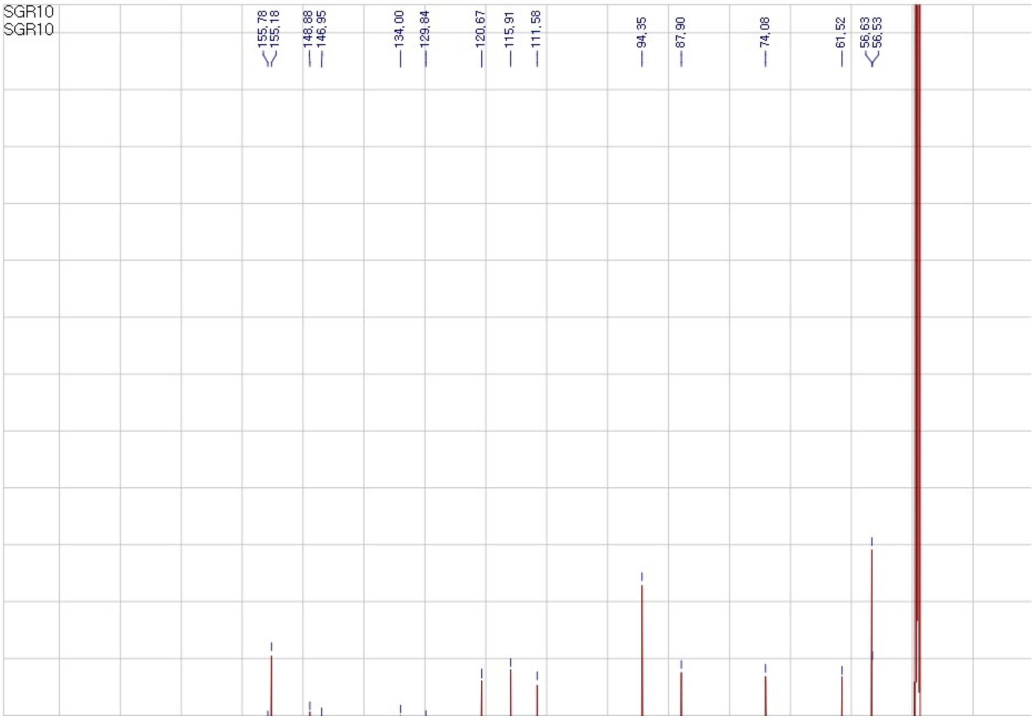

3. The ^{1}H NMR spectrum of ***2***

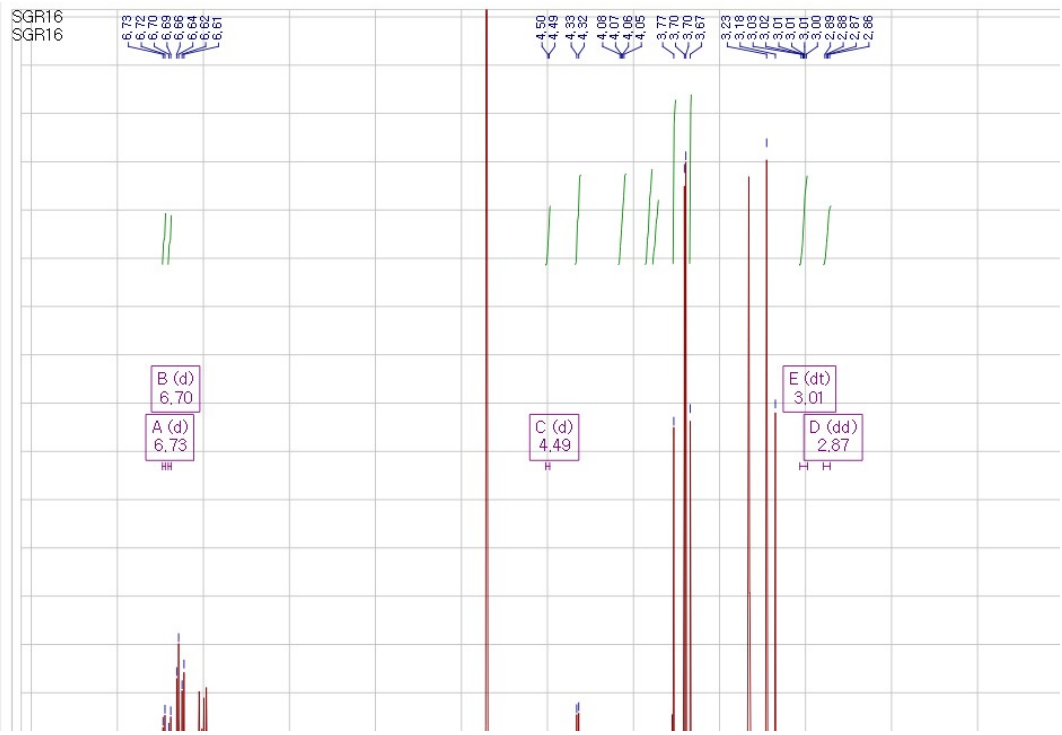

4. The ^{1}H NMR spectrum of ***3***

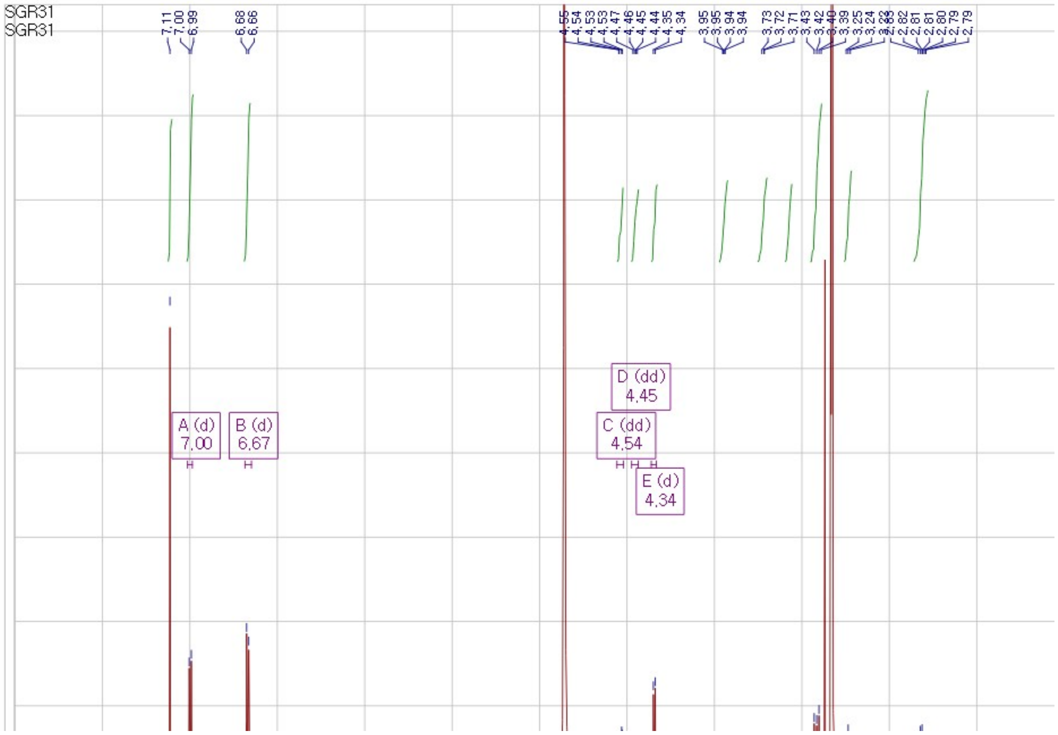

5. The ^{13}C NMR spectrum of ***3***

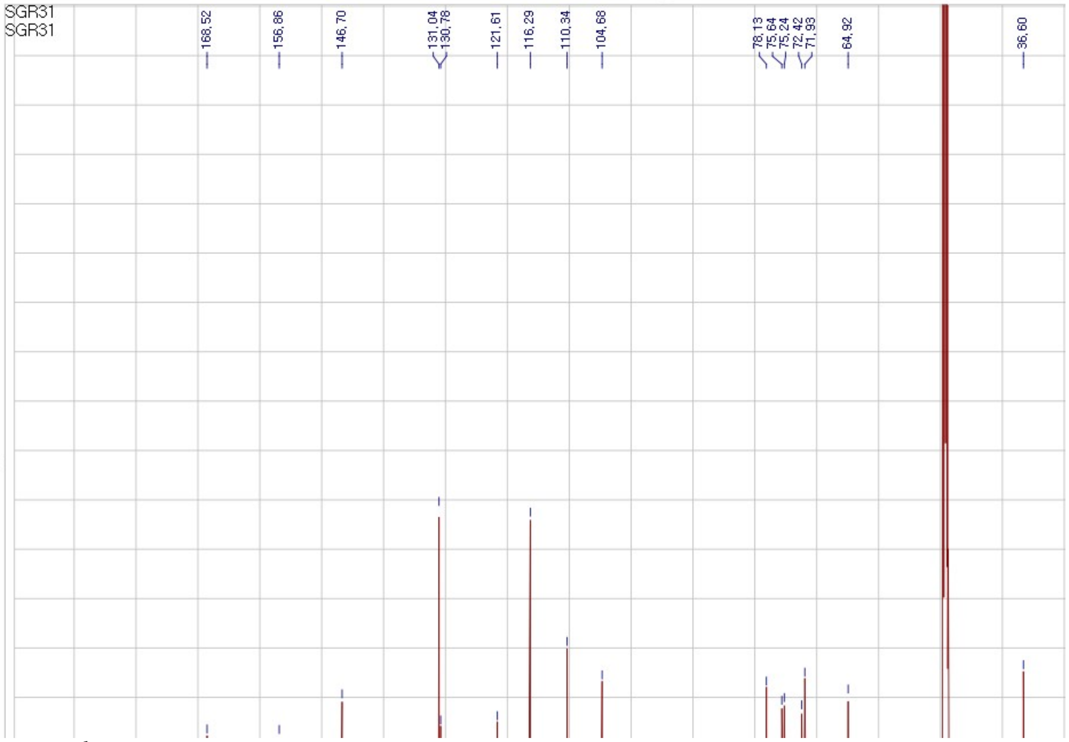

6. The ^{1}H NMR spectrum of ***4***

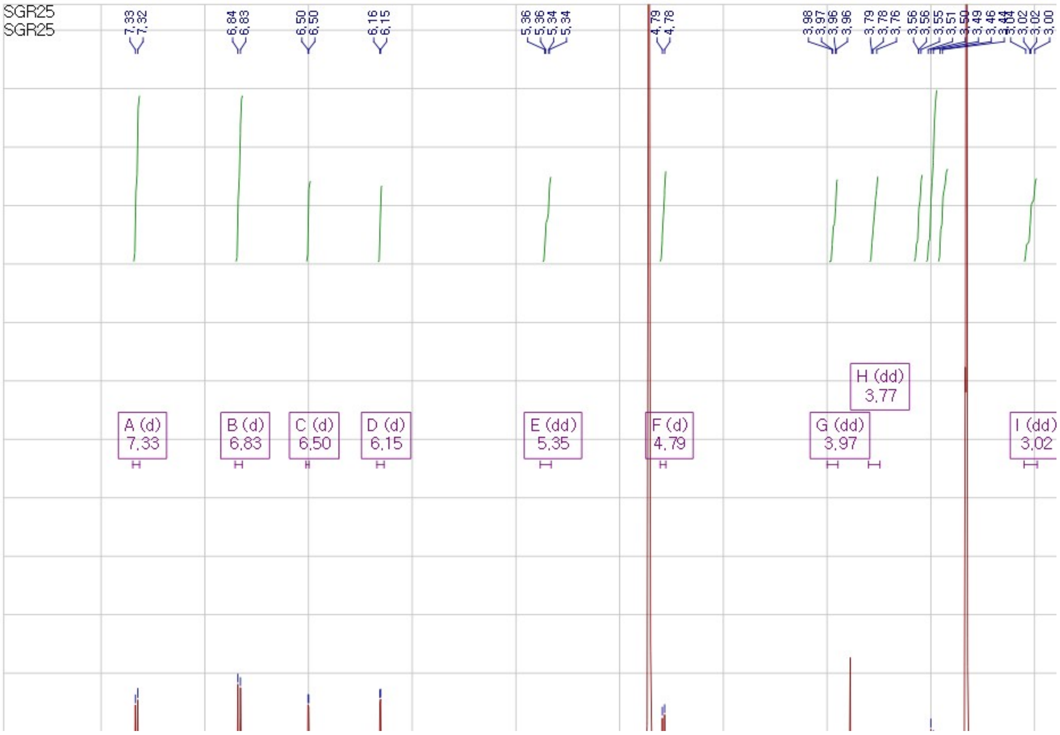

7. The ^{13}C NMR spectrum of **4**

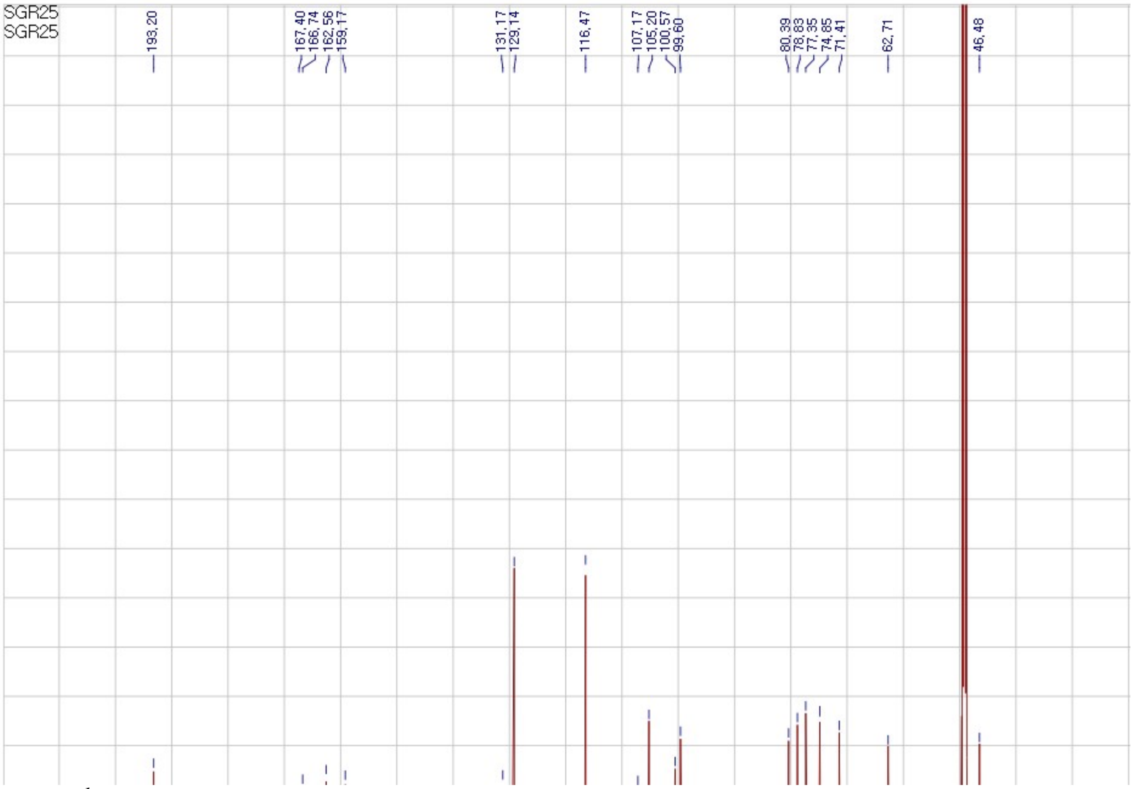

8. The ^{1}H NMR spectrum of **5**

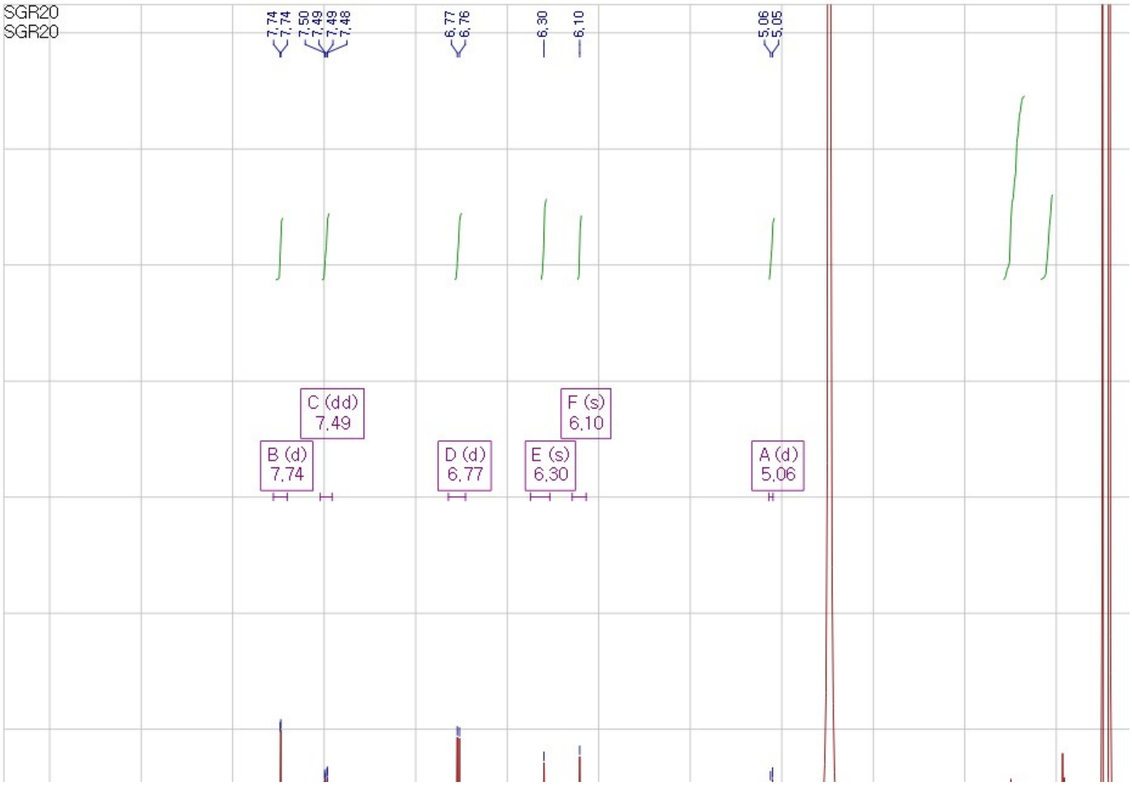

9. *The ^{13}C NMR spectrum of* ***5***

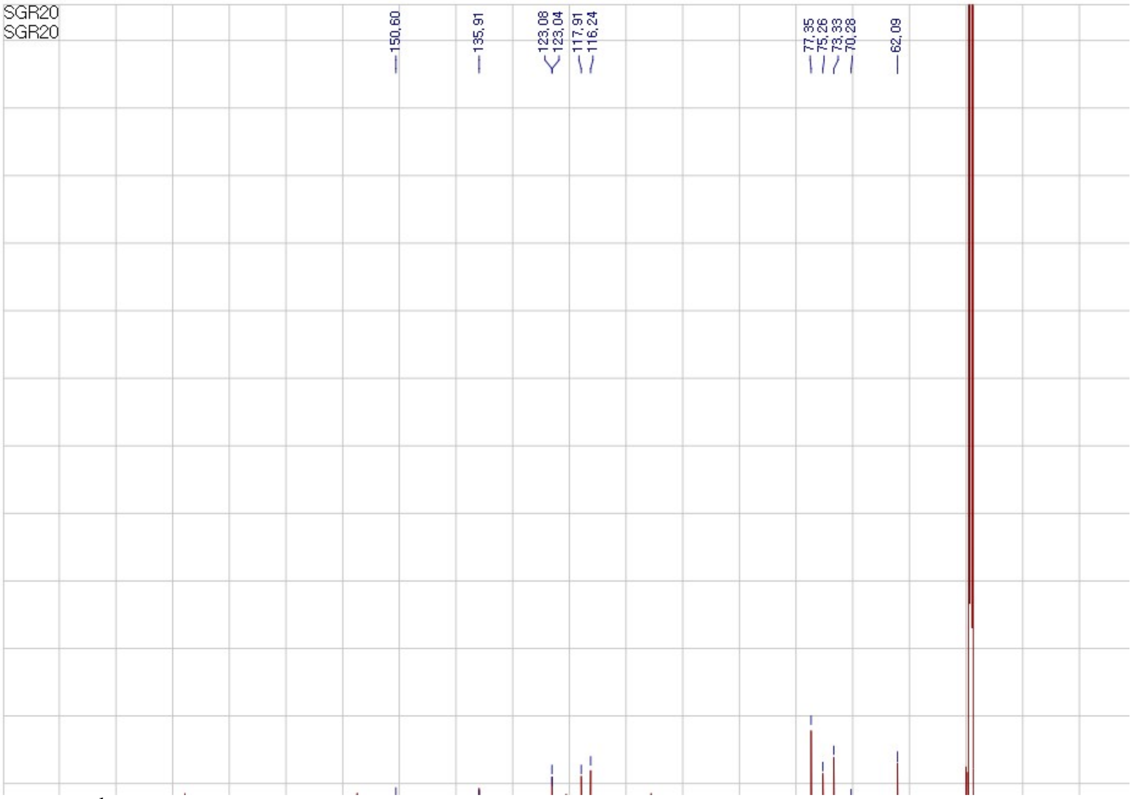

10. *The ^{1}H NMR spectrum of* ***6***

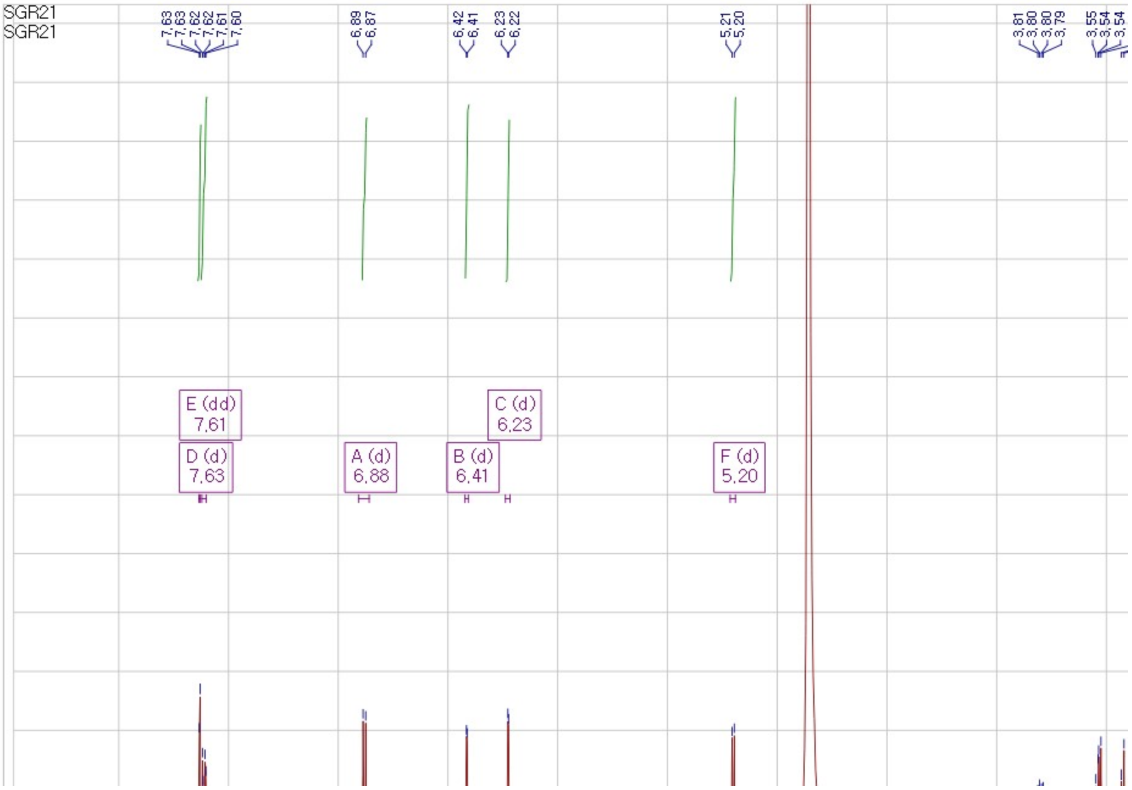

11. *The* [13]*C NMR spectrum of* ***6***

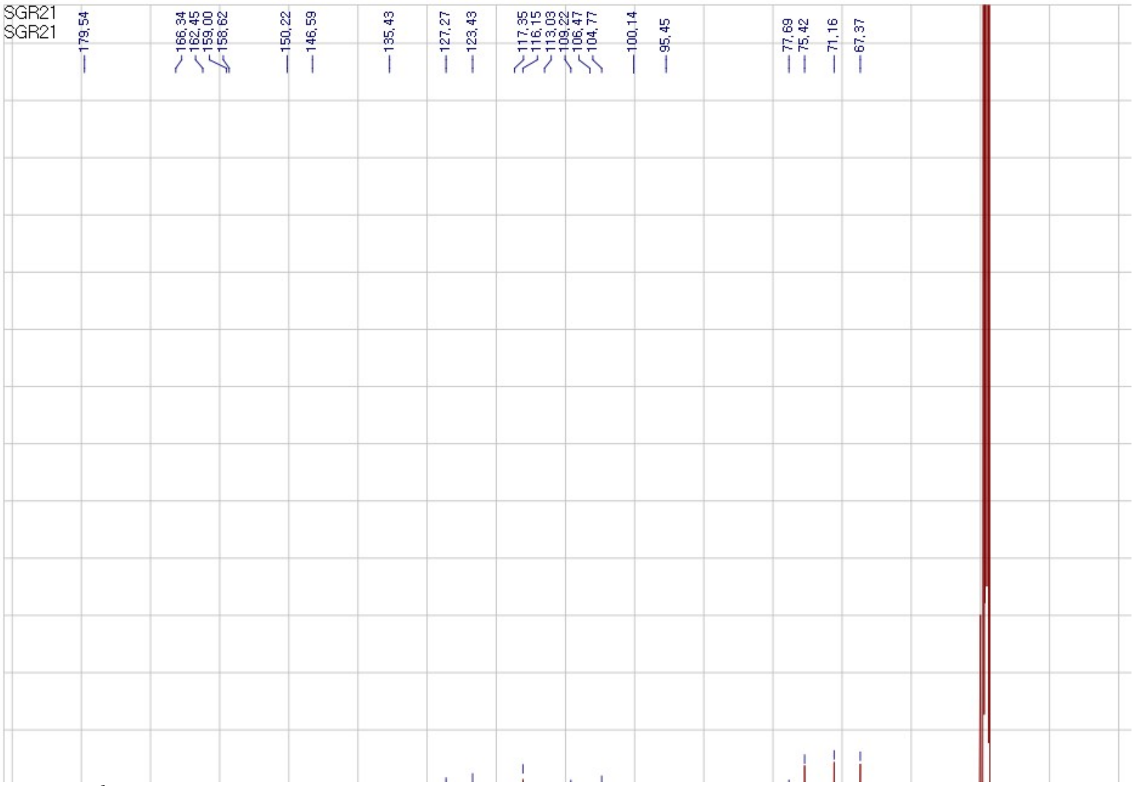

12. *The* [1]*H NMR spectrum of* ***7***

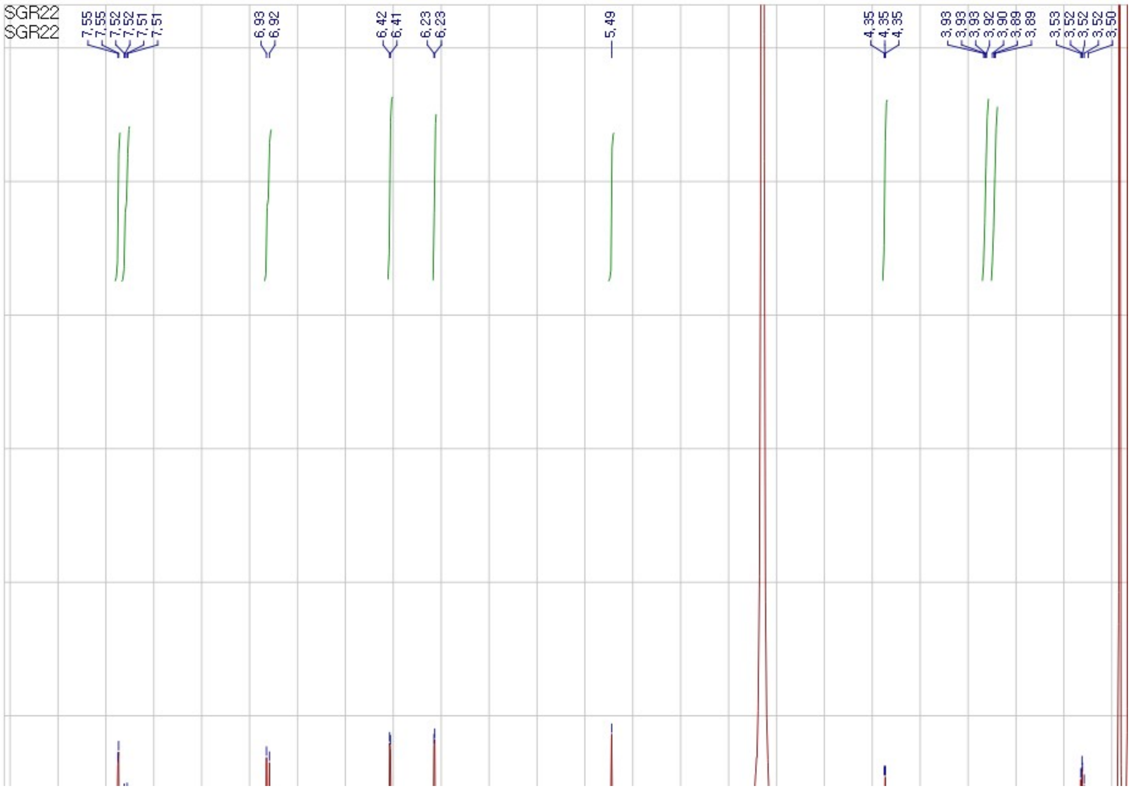

*13. The ^{13}C NMR spectrum of **7***

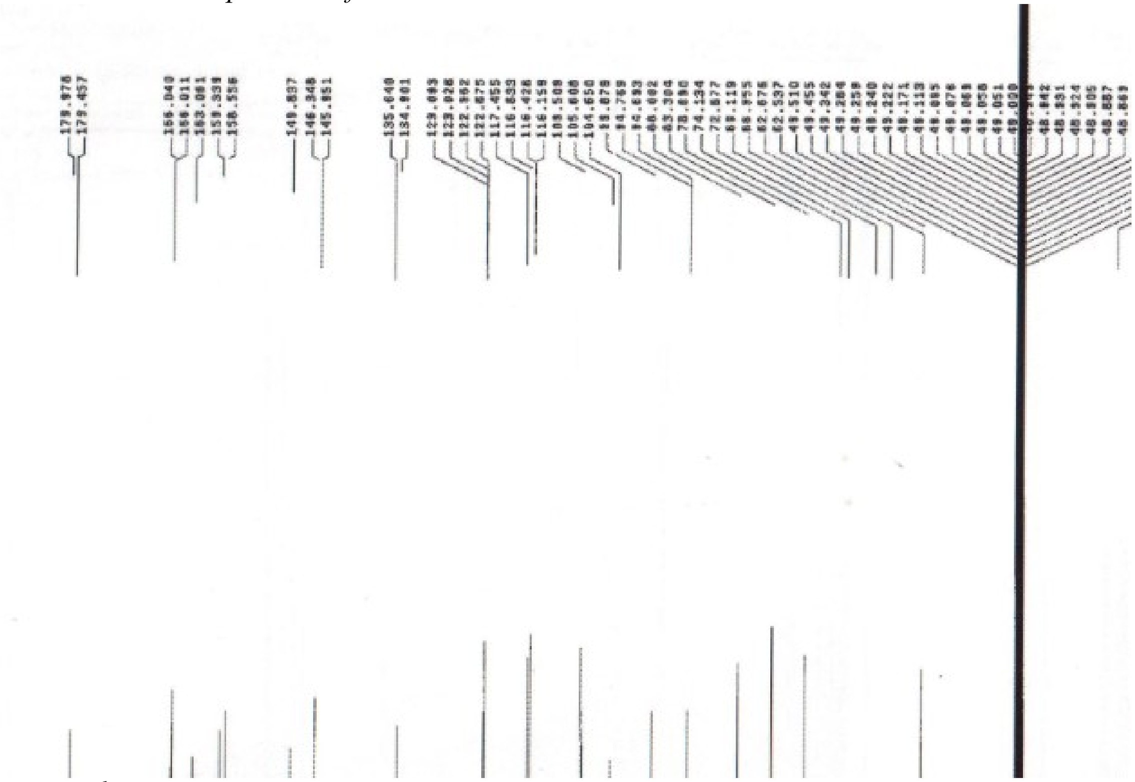

*14. The ^{1}H NMR spectrum of **8***

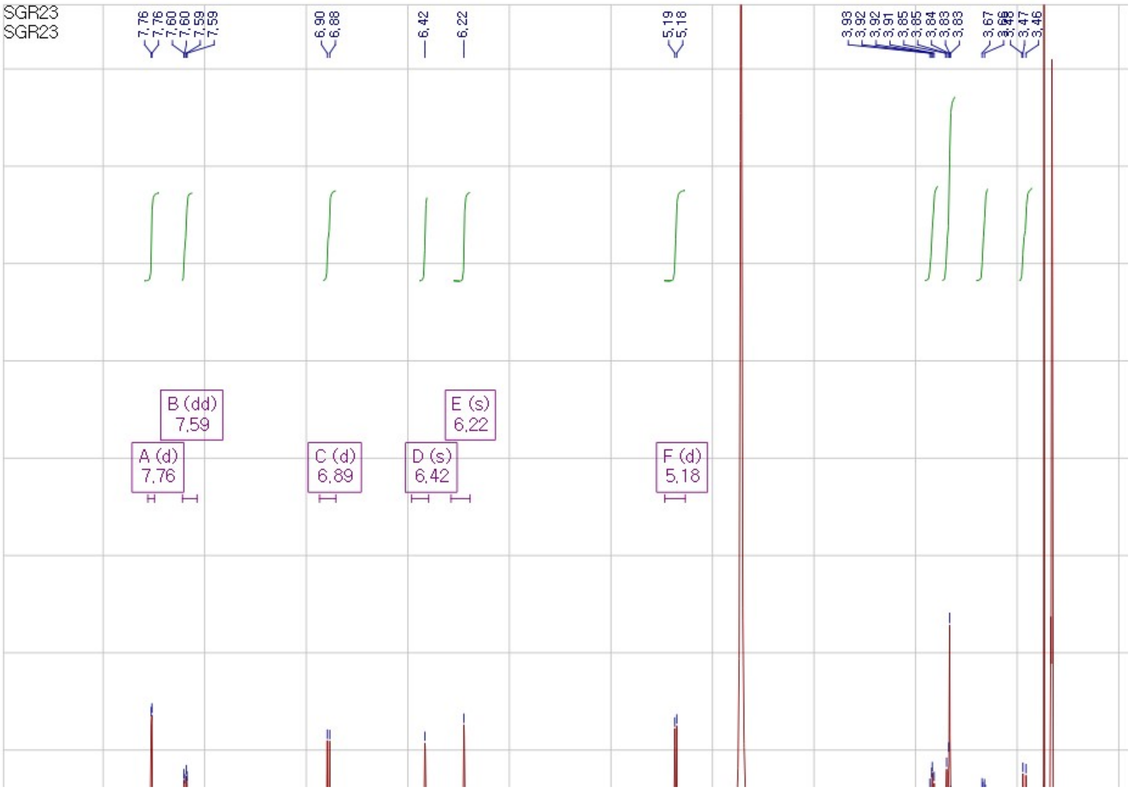

15. The ^{13}C NMR spectrum of ***8***

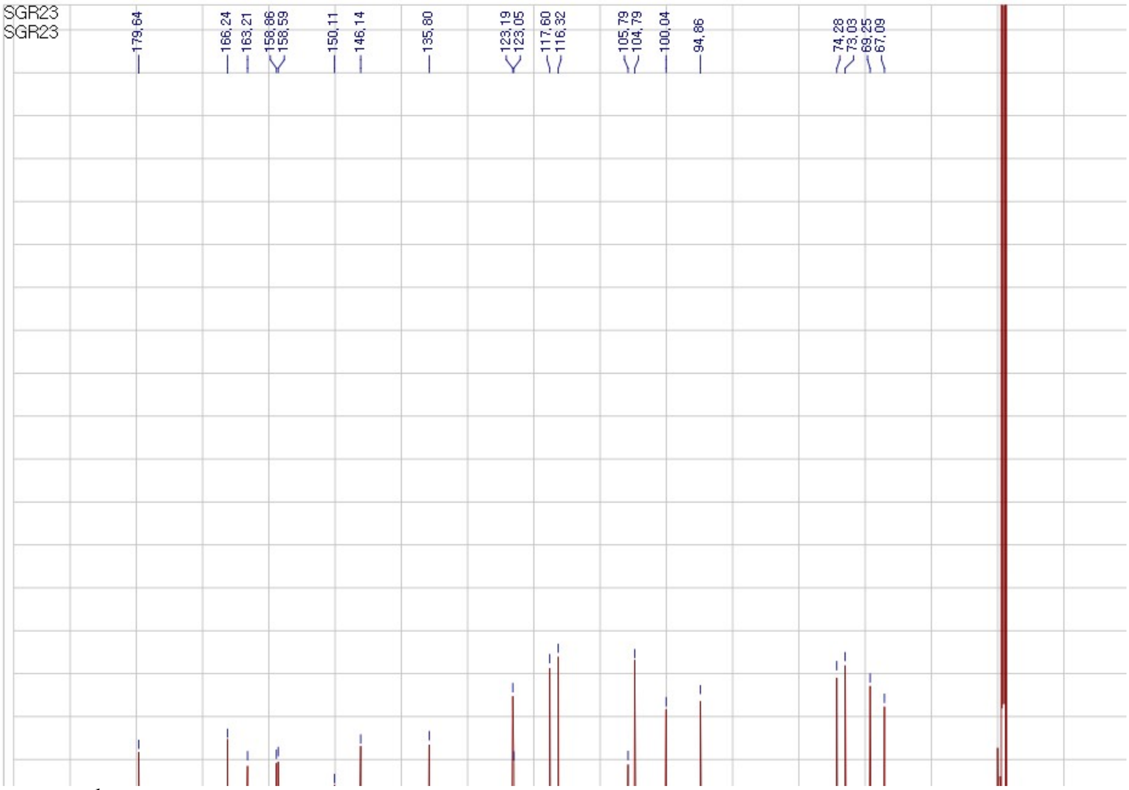

16. The ^{1}H NMR spectrum of ***9***

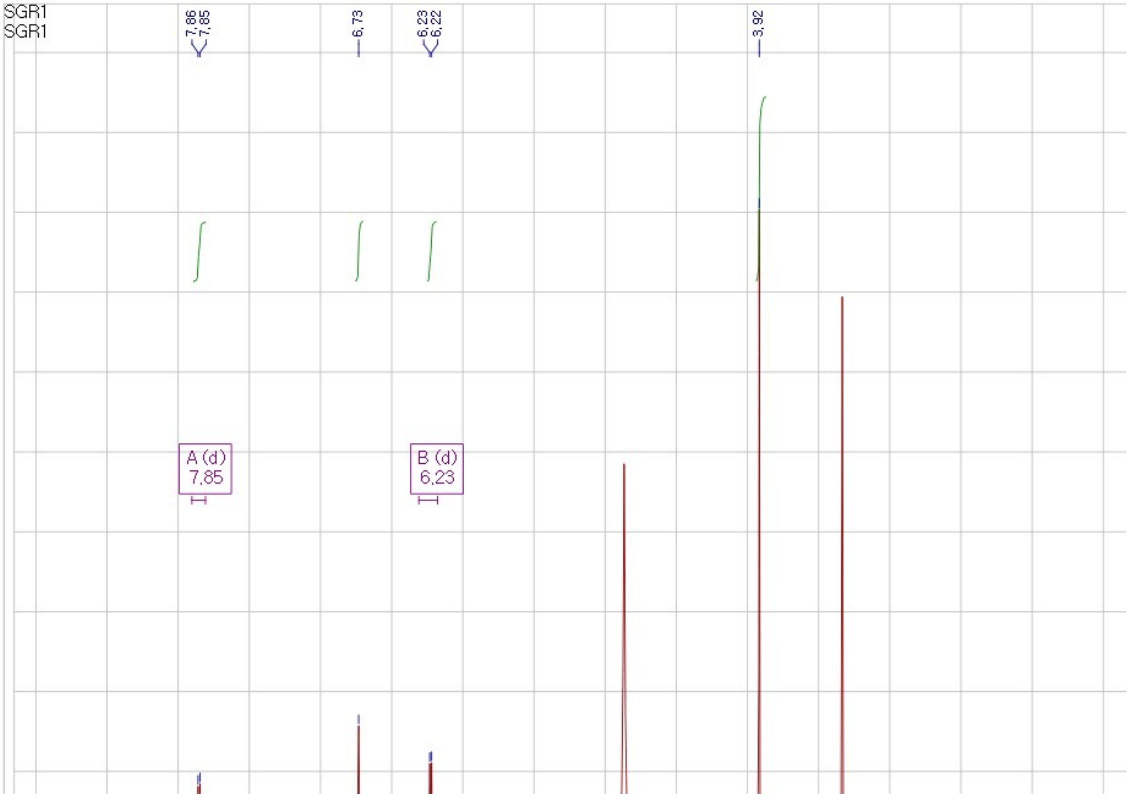

*17. The ^{13}C NMR spectrum of **9***

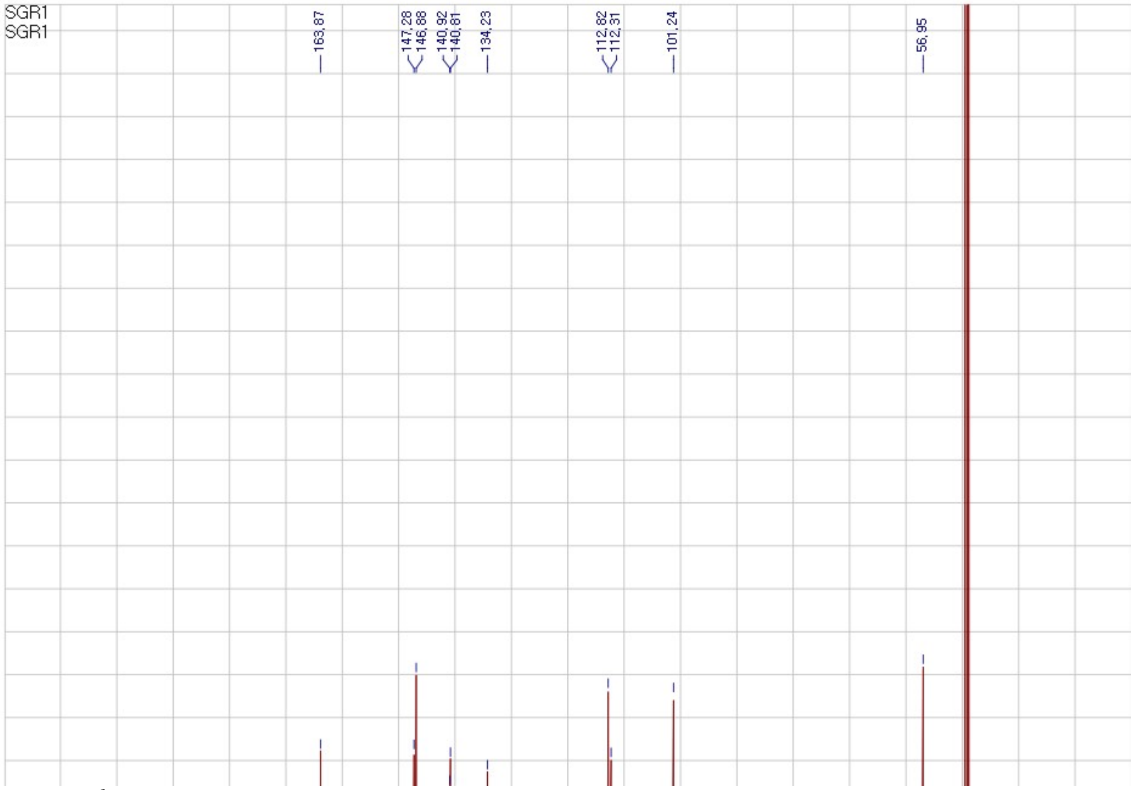

*18. The ^{1}H NMR spectrum of **10***

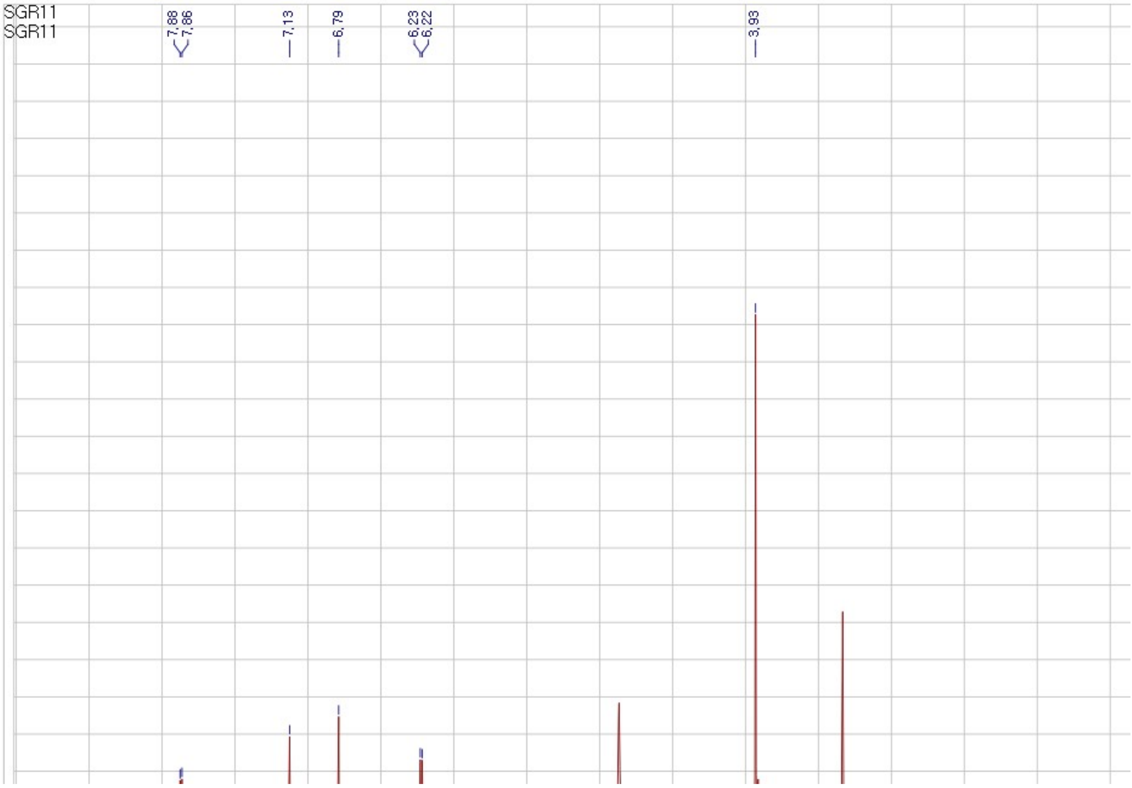

19. The ^{1}H NMR spectrum of ***11***

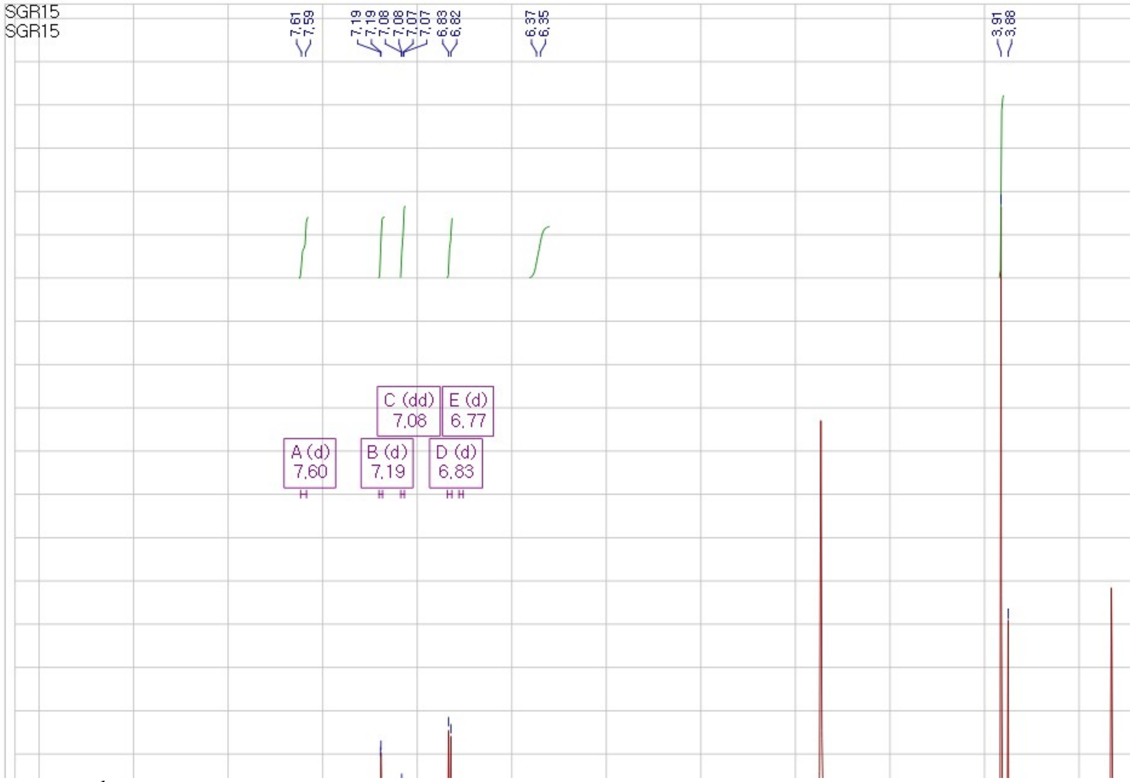

20. The ^{1}H NMR spectrum of ***12***

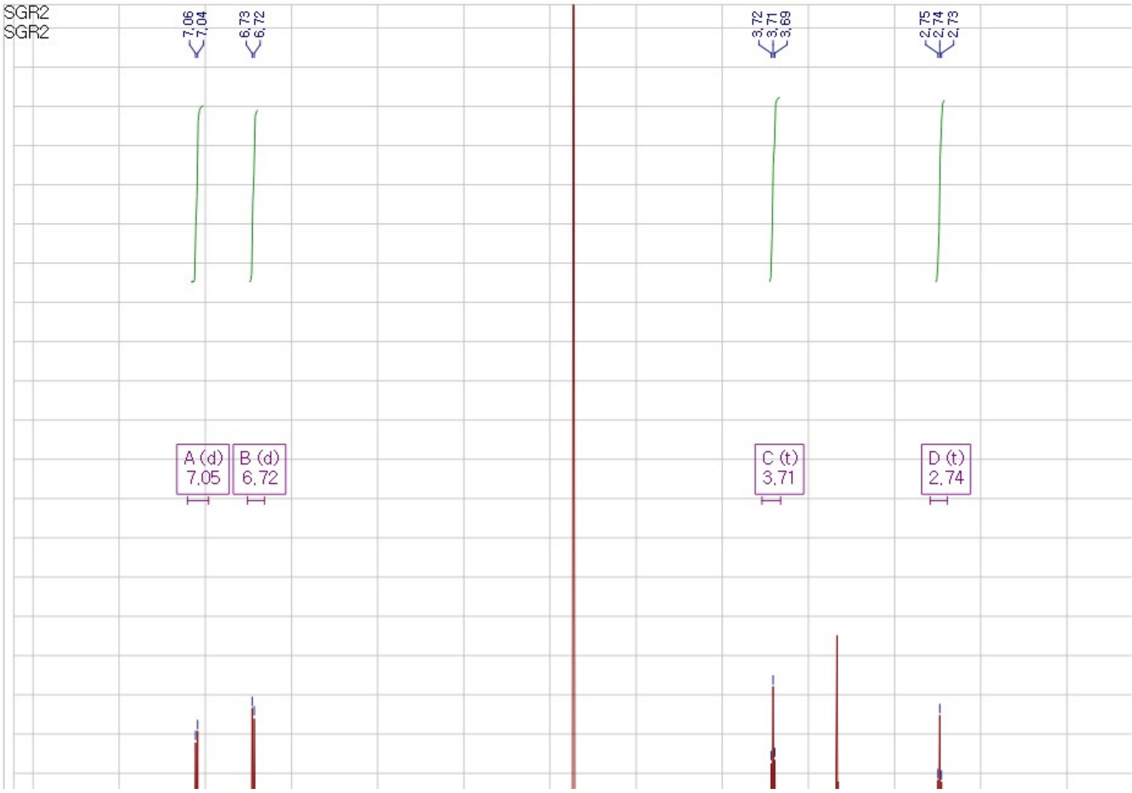

*21. The ^{1}H NMR spectrum of **13***

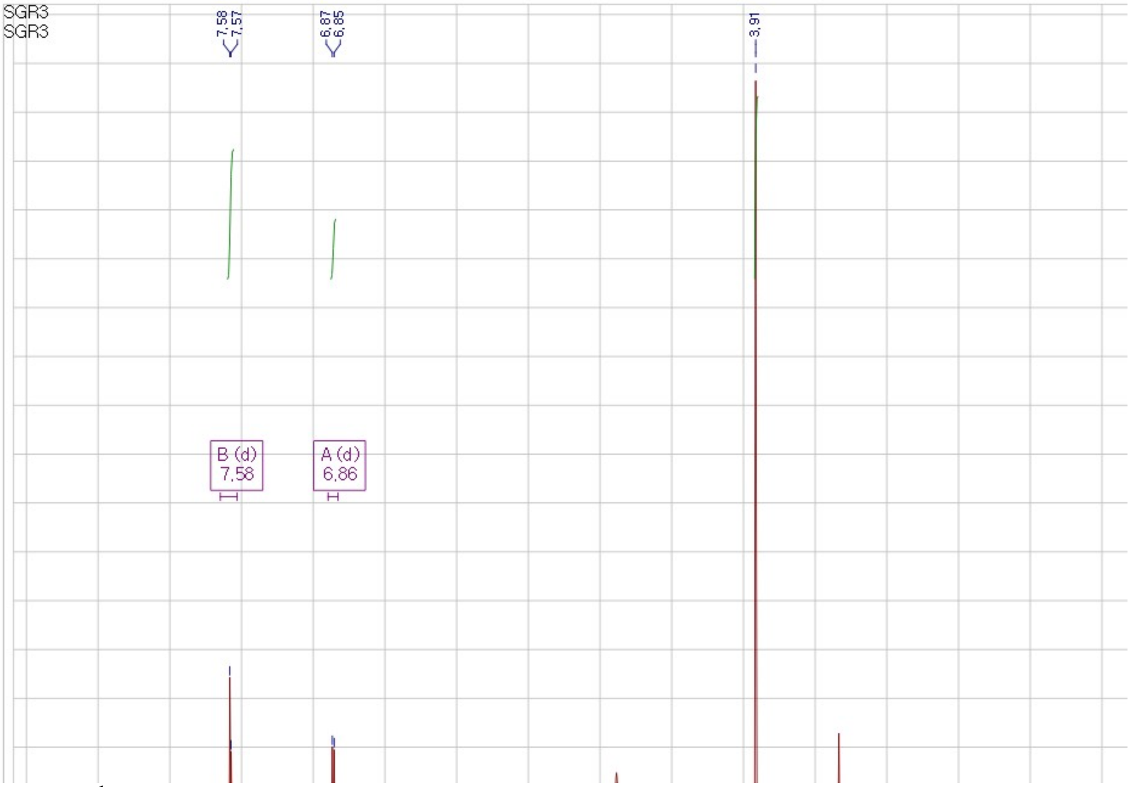

*22. The ^{1}H NMR spectrum of **14***

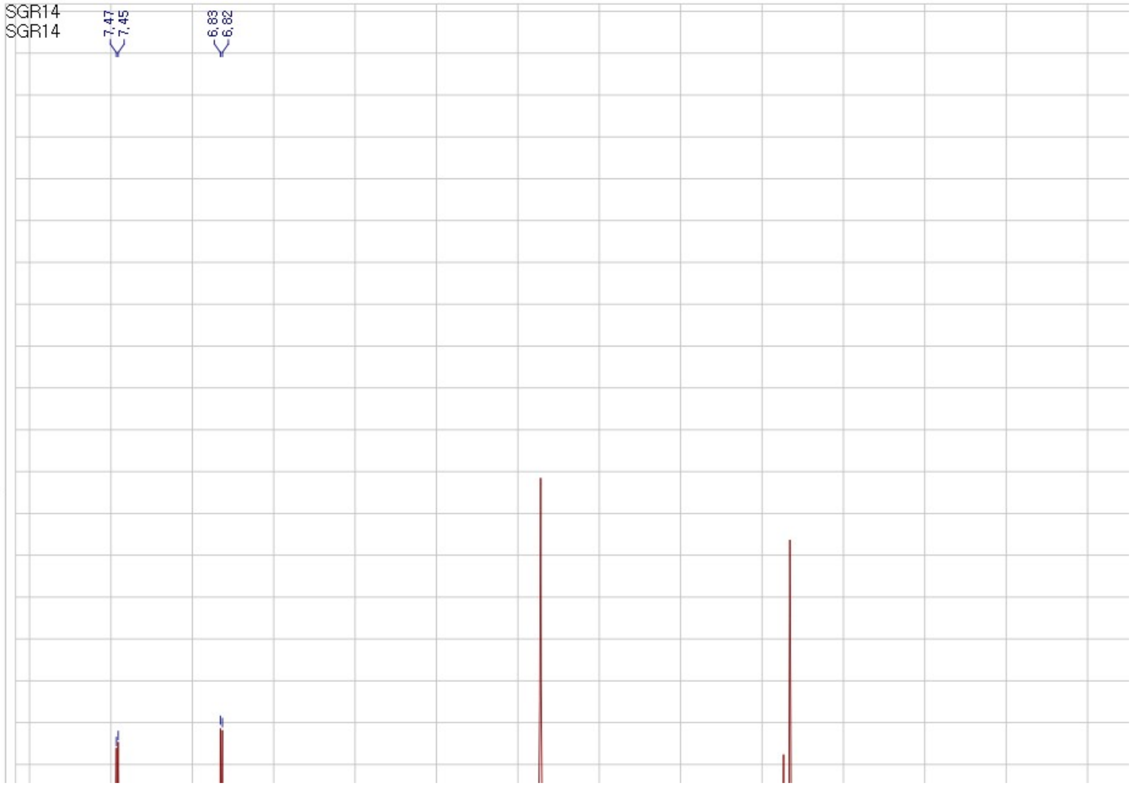

23. The ^{1}H NMR spectrum of ***15***

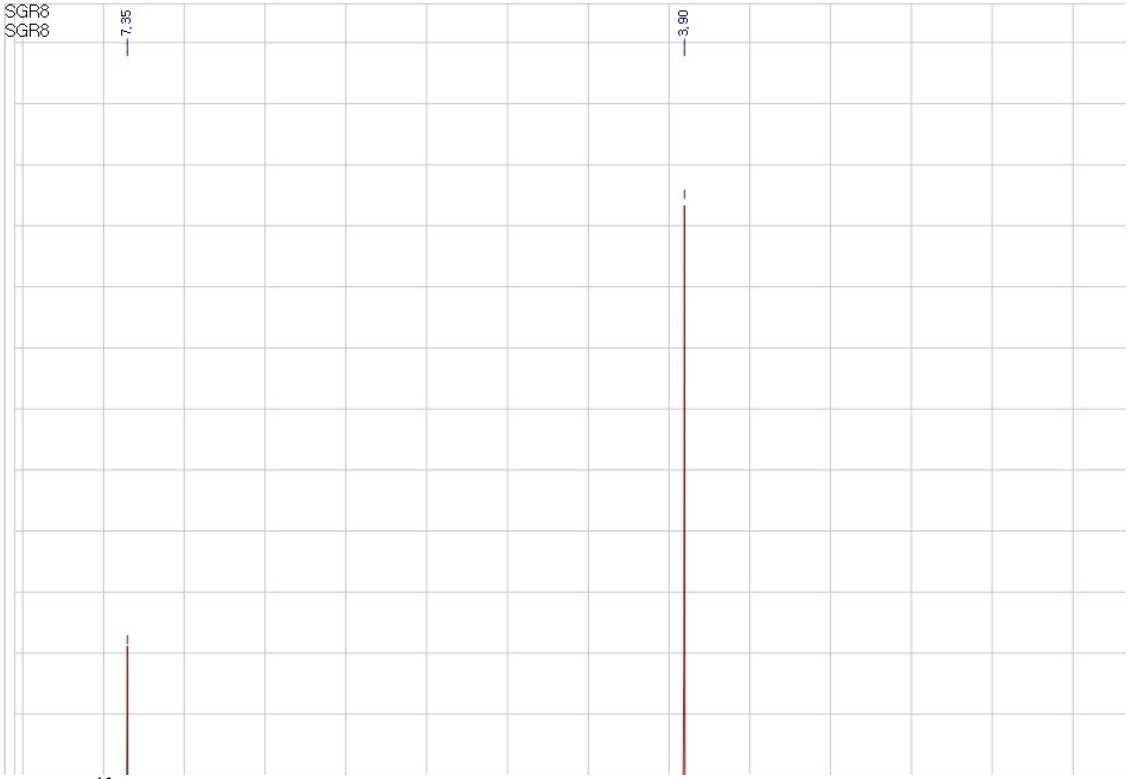

24. The ^{13}C NMR spectrum of ***15***

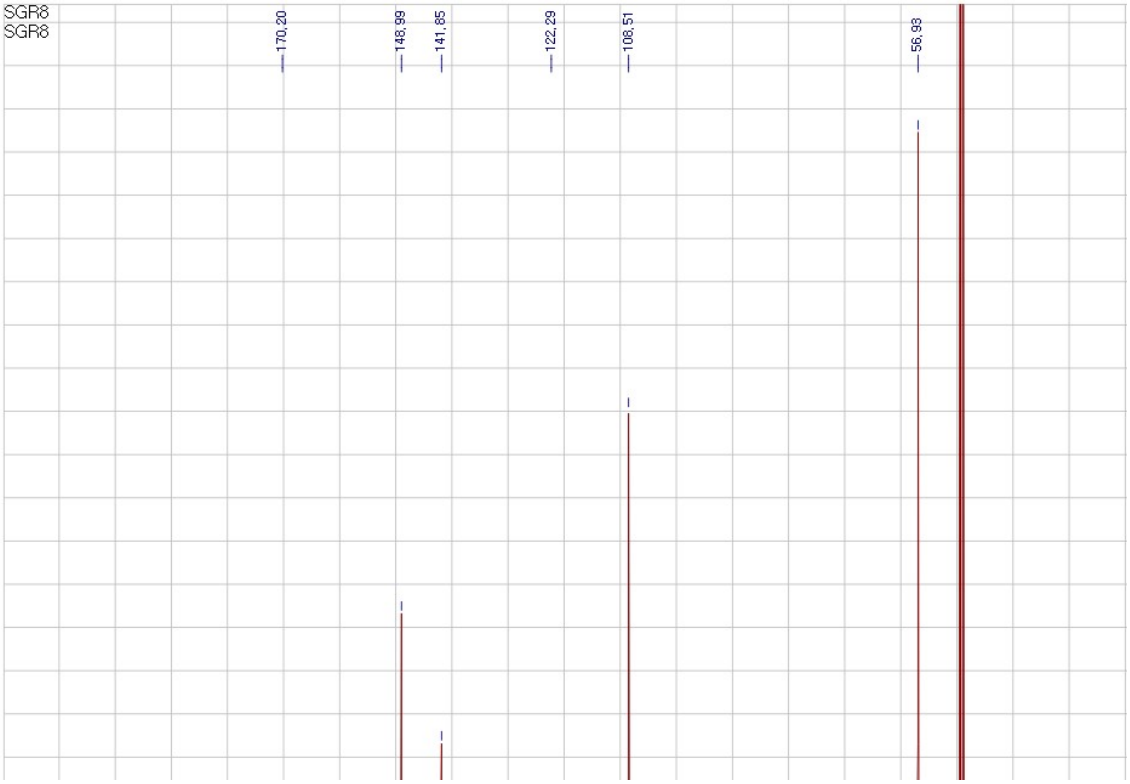

Supplement: Supplementary file 1 [file ijms-20-05832-s001.pdf]
